# MicroRNAs as Biomarkers for Animal Health and Welfare in Livestock

**DOI:** 10.3389/fvets.2020.578193

**Published:** 2020-12-18

**Authors:** Silvia Miretti, Cristina Lecchi, Fabrizio Ceciliani, Mario Baratta

**Affiliations:** ^1^Department of Veterinary Sciences, University of Torino, Grugliasco, Italy; ^2^Department of Veterinary Medicine, Università degli Studi di Milano, Milan, Italy

**Keywords:** microRNA, biomarker, health, welfare, ruminants, pig, poultry, livestock

## Abstract

MicroRNAs (miRNAs) are small and highly conserved non-coding RNA molecules that orchestrate a wide range of biological processes through the post-transcriptional regulation of gene expression. An intriguing aspect in identifying these molecules as biomarkers is derived from their role in cell-to-cell communication, their active secretion from cells into the extracellular environment, their high stability in body fluids, and their ease of collection. All these features confer on miRNAs the potential to become a non-invasive tool to score animal welfare. There is growing interest in the importance of miRNAs as biomarkers for assessing the welfare of livestock during metabolic, environmental, and management stress, particularly in ruminants, pigs, and poultry. This review provides an overview of the current knowledge regarding the potential use of tissue and/or circulating miRNAs as biomarkers for the assessment of the health and welfare status in these livestock species.

## Introduction

Monitoring animal welfare is challenging. Keeping farm animals clinically healthy, without disease or distress, is fundamental for the production of safe and high-quality food. This topic is highly relevant both for governments and for food industries worldwide. Furthermore, consumers pay increasing attention to how animals are reared and, consequently, to how animal-derived food products are obtained. The concept of animal welfare includes a great variety of aspects. Recently, the Welfare Quality protocol introduced the use of animal-based measures that are focused on animal needs and include the evaluation of appropriate (valid, reliable, and viable) indicators that allow the assessment of the physical and mental welfare of animals (1). Animal-based measures are particularly useful because they show the effects of interaction between the animal and its environment. Impaired animal welfare is often caused by chronic stress resulting from an inability to cope with environmental factors combined with genetic vulnerability (e.g., the concentration of neurotransmitters such as serotonin and individual immune response capacity) (2–4). According to the current literature, livestock welfare indicators are classified into three main categories, namely, physiological measures, behavioral observations, and product quality (5–8). Physiological measures, including blood parameters (9–11) and behavior, allow the assessment of animal welfare *in vivo* (12). Systemic metabolic perturbation resulting from chronic stress has been also investigated using metabolomics, and has led to the identification of parameters directly associated with management and housing conditions and regulated by the hypothalamic–pituitary–adrenal axis (HPA) (13, 14). Other studies have aimed to identify hormones and other molecules that are quantified at levels out of a “physiological range” (15–17).

miRNAs are fine-tuning coordinators of cellular processes, including the modulation of animal development, homeostasis, immune responses, and control of infection, and are also crucial for the regulation of stem cell self-renewal and tissue differentiation (18–21). Following a physiological stimulus or injury, circulating miRNAs (c-miRNAs) can be released from cells into the blood or other body fluids in either an active (secretion) or passive (membrane leaking) manner (22–24). Much of the interest generated by c-miRNAs relates to their involvement in the regulation of molecular pathways of recipient cells and their remarkable potential as easily accessible biomarkers of diseases and disorders (25).

## miRNAs as Potential Biomarkers

Some of the most intriguing and potentially interesting aspects in identifying miRNA molecules as biomarkers derive from their highly regulated spatial and temporal expression, their active secretion from cells into the extracellular environment, and their high stability in body fluids (26). C-miRNA profiles change under conditions such as diseases, viral or bacterial infections (27–29), and physiological states (e.g., pregnancy) (30, 31), indicating that c-miRNAs are suitable biomarkers for monitoring different physical conditions in animals.

Emerging evidence from rodent models has indicated that c-miRNAs may also serve as biomarkers of resilience or vulnerability to stress (32). The dysregulated expression of miRNAs has been investigated as markers of a variety of diseases, including mental disorders such as anxiety (33). Recent studies have indicated that miRNAs contribute to numerous aspects of neurogenesis, neural plasticity, and the stress response, while also modulating the expression of genes involved in chronic psychosocial stress in both humans and rodents (34, 35). The identification of stress and the relationship between stressful conditions and their psychological causes is particularly challenging in farm animals; nevertheless, chronic stress induced by social conflict, social isolation, and overcrowding has also been reported (36–38). To the best of our knowledge, no study to date has investigated the role of miRNAs in the molecular mechanisms underlying mental stress responses in livestock species. Given that most livestock species are social, the ability of miRNAs to modulate the molecular networks associated with mental stress merits further investigation.

Usually, the need to adapt the technologies developed for human medicine to animal species is one of the main reasons for the delayed progress in veterinary science. Owing to the high level of sequence homology across species, miRNAs can be readily detected without the need for protein-specific antibodies and species-specific assays. Therefore, the identification of suitable miRNAs is an interesting field of research that may provide an in-depth and complete overview of animal welfare and related health conditions at the molecular level. C-miRNAs are considered among the most promising clinical biomarkers for the identification of stress-related disorders in animals, becoming valid tools to assess the welfare of an animal throughout its life, while also having the potential to allow the scoring of the quality of animal products along the food supply chain.

The concept of stress was recently proposed to be restricted to “conditions where an environmental demand exceeds the natural regulatory capacity of an organism, in particular situations that include unpredictability and uncontrollability,” and must be strictly related to a health condition (39). Several challenging events during the productive life of livestock species may be included within these “unpredictable and uncontrollable” situations, including immunological and metabolic stress, management stress, stress due to the splitting and regrouping of animals during their production cycle, weaning stress, stress associated with diet changes, handling or transport stress, and environmental stress.

This review aimed to describe and critically assess the relevant current literature regarding these challenging events, giving only a brief overview of miRNAs involved in immune responses induced by infectious diseases. Despite the increased interest in investigating miRNAs involved in veterinary-relevant infectious diseases in the past few years, this topic is not within the main scope of this review, and has been described in excellent recent reports (19, 20, 25, 40–44) (Supplementary Table 3).

## miRNAs Related to Health and Welfare in Cattle

**Figure 1:**
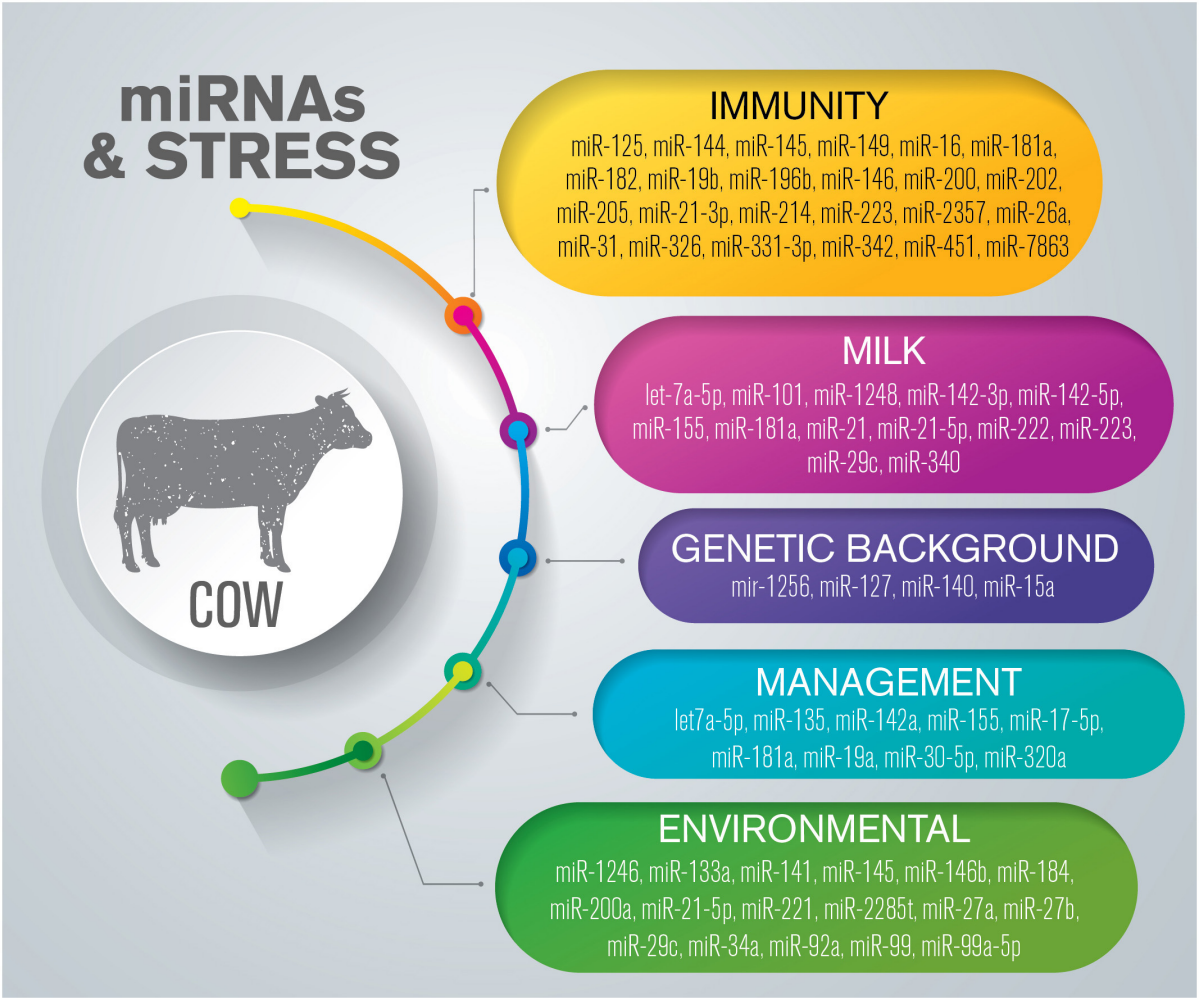
Overview of miRNAs dysregulated in response to stress in cattle. The list is limited to miRNAs confirmed by sequencing and validated through molecular approaches such as RT-qPCR and *in situ* hybridization.

Acute and chronic events exert different influences on bovine metabolic pathways, and can affect the quality of life and productivity of these animals. Immunity, mammary gland health, milk composition, and metabolic parameters are regulated by several molecular networks, which include miRNAs (Figure 1) (Supplementary Table 1).

### Immunity

Diseases, disorders, and other stressors deriving from environmental or management conditions can suppress the immune system through the activation of the HPA axis and the sympathetic–adrenal–medullary (SAM) system (45). Recent studies have proposed that miRNAs play crucial roles in bovine immunity by regulating different immune cell functions (41, 46). In particular, miRNAs seem to be interesting as candidate biomarkers to detect changes in immune mechanisms involved in mycobacterial infections and mastitis.

A recent review defined miRNAs as key regulators of host gene expression and immune defenses, as well as promising biomarkers for resistance to mycobacterial infection (20). In cattle, many miRNAs have been reported to be modulated during mycobacterial infections (47–51). The expression of miR-19b, miR-196b, and miR-146, which are immune- and inflammation-related c-miRNAs, is altered during infection caused by *Mycobacterium bovis* and *M. avium* subspecies *paratuberculosis*, and have been proposed as potential biomarkers for these diseases (50, 51).

Mastitis is one of the most frequent and economically important diseases in the dairy sector, and greatly threatens the welfare of affected animals. Several studies have investigated the role of miRNA in mastitis induced by *Streptococcus uberis, Staphylococcus aureus*, and *Escherichia coli* (52–54). These studies mostly aimed to clarify the role of miRNAs in innate immune responses and identify candidate genes and miRNAs suitable for use in developing strategies for the prevention, diagnosis, or treatment of mastitis (52, 53, 55). Using RT-qPCR, Naeem et al. investigated several immune-related miRNAs in *S. uberis*-infected bovine mammary tissue, and found that miR-181a, miR-16, and miR-31 were significantly downregulated, whereas miR-223 was upregulated, in infected tissue when compared with healthy tissue (56). Several years later, the miRNome and transcriptome of milk and blood CD14+ monocytes collected from Holstein Friesians cows experimentally infected with *S. uberis* were characterized for the first time using next-generation sequencing (NGS) (57). The authors identified several differentially expressed (DE) miRNAs, previously described as targeting immune or inflammatory regulators in other species and bovine mammary epithelial cells, at different time points (41, 57). Their results showed that miR-223 was upregulated, as previously reported by Naeem et al. (56), but in contrast, miR-146 was downregulated (56, 57). Interestingly, this study highlighted that most of the downregulated miRNAs expressed in milk monocytes isolated from infected animals, such as miR-149, were predicted to preferentially target genes involved in innate immunity, including those involved in the Toll-like receptor (TLR), NOD-like receptor (NOD), and the RIG-I-like receptor (RGI-I) signaling pathways. The transcriptional suppression of these miRNAs enables the activation and amplification of pro-inflammatory responses (57).

Another pathogen frequently involved in mastitis etiology is *S. aureus*. Using RNA-seq analysis, Fang et al. demonstrated the pivotal roles of miR-223 and miR-21-3p in defending the host against bacterial infection through their immunoregulatory activity on innate immune-related genes (e.g., *CXCL14* and *KIT*) (58). By combining the results of high-throughput sequencing, customized miRNA chip data, and miRNome analysis, another group characterized the expression of genes in mastitis-infected tissue during the late stage of natural infection with *S. aureus* (59). MiR-26a, predicted to target a bridging molecule (FGA) involved in host defense mechanisms against *S. aureus*, was downregulated in infected tissue when compared with that of normal samples, thereby confirming the role of miR-26a in regulating tissue defense (59).

A recent study investigated the roles of miRNAs in host defenses against *S. aureus* and *E. coli* by comparing the two models of bovine mastitis infection (52). Deep sequencing and a comparative analysis of infected vs. healthy tissue, and between tissues infected by each of the pathogens, led the authors to propose miR-7863 as a biomarker for *S. aureus*-induced mastitis and miR-202 and miR-2357 as biomarkers for *E. coli*-induced mastitis. MiR-144 and miR-451 were defined as being useful for discriminating between the two pathogens because they were significantly upregulated in *S. aureus*-infected mammary glands, and markedly downregulated in those infected with *E. coli* (52).

Relatively few reports have described the profile of blood miRNAs of dairy cows affected by mastitis (54, 57, 60). Several miRNAs have been identified as being DE in the serum of cattle affected by *S. aureus*-related mastitis compared with healthy animals. Among them, miR-144 and miR-125, which are involved in the regulation of immune responses through signaling pathways such as TLR, TGF-beta, and MAPK, were found to be upregulated (54). Recently, the expression profile of miRNAs in the peripheral blood of cattle with *E. coli*-related mastitis was characterized by NGS and RT-qPCR (60). The authors identified several immune-related miRNAs that were DE, some of which were upregulated (miR-200, miR-205, miR-182, miR-214, and miR-145) and some downregulated (miR-342, miR-326, and miR-331-3p). KEGG pathway enrichment analysis showed that the DE-miRNAs were preferentially involved in cytokine–cytokine receptor interaction, chemokine signaling pathway, leukocyte transendothelial migration, T cell receptor signaling pathway, TLR signaling pathway, and cell adhesion molecules, thereby confirming their role in innate immunity and inflammatory responses (60). These DE-miRNAs merit further investigation as potential biomarkers in *E. coli*-induced mastitis.

Different authors have reported that miR-223 is highly expressed in mammary gland tissue. This miRNA may play a pivotal role in regulating inflammatory responses against pathogens, and may potentially represent a candidate diagnostic marker for bovine mastitis (56–58, 61, 62). In humans, miR-223 is essential for innate immune and inflammatory responses governing neutrophil proliferation, activation, and granulopoiesis (63, 64). Neutrophils are the first effectors of the inflammatory response triggered by mastitis infection and represent an important line of defense against pathogenic microorganisms. It would be of interest to explore if miR-223 would similarly affect neutrophils in cattle.

### miRNAs and Milk

Importantly, calves lack circulating antibodies at birth. In the first days of life, the ability of newborns to fight pathogens is completely dependent on the passive immunity provided by ingested colostrum (65). Colostrum contains important nutrients, bioactive components, and higher amounts of miRNAs than mature milk. The possible effects of colostrum ingestion on calves have been recently reviewed (66).

miRNAs in milk may be actively secreted by the mammary gland (67) or passively leaked by mammary gland cells (68), and their expression profiles differ between colostrum and milk (69), as well as among cattle breeds (67). Through a systematic sequencing-based analysis of colostrum and raw milk at various stages of lactation, Chen *et al*. identified miRNAs involved in the immune response and immune system development, such as miR-181a, miR-155, and miR-223, that showed markedly higher abundance in colostrum than in milk (69). Immune-related miRNAs were also identified in milk-derived microvesicles. A recent study compared the miRNAs in milk and colostrum exosomes of Holstein and native Turkish Dogu Anadolu Kirmizisi (DAK) cows, a breed extremely resistant to harsh environmental conditions (67). Through sequencing and a second validation step using RT-qPCR, miR-142-3p, miR-29c, miR-222, miR-1248, let-7a-5p, miR-340, miR-101, and miR-21-5p were found to be DE between the two breeds. Functional enrichment analysis revealed that the total number of miRNAs in the colostrum of DAK cows was more related to immunological pathways compared with that in milk (67). The abundance and composition of miRNAs in milk and colostrum may also be influenced by management-related factors, including nutrition (70) and subclinical disorders (61, 62). The amount of inflammation-related miRNAs in the milk of cows affected by mastitis can vary, suggesting that they could potentially be used to detect bovine mastitis, as speculated for miR-222 and miR-21 by Lai et al. and miR-223 and miR-142-5p by Cai et al. (61) and Lai et al. (62). Milk miRNAs may potentially be used for monitoring the welfare of cows and preventing possible disorders in the health of calves; however, further research is needed to elucidate their reliability as biomarkers.

### miRNAs and Genetic Background

In cattle, stressor stimuli-related miRNAs are influenced by the genetic background (71). Ioannidis et al. investigated the potential application of c-miRNAs as markers for genetic attitude and performance in dairy cattle, including aging, fertility, and welfare traits (71). The most pronounced changes in c-miRNA levels among calves, heifers, and cows were found during the early stage of life. Most miRNAs, such as miR-127 or miR-140, were associated with health traits (mastitis, fertility, lameness) and were linked to immune responses and inflammation. Twelve age- and one genetic line-related (miR-15a) miRNAs were reported to be DE (71). Plasma miRNA levels were associated with longevity indicators such as telomere length, milk production and composition, milk somatic cell count, fertility, lameness, and blood metabolites linked to body energy balance and metabolic stress (71). Dystocia and perinatal mortality are quantitative traits that significantly influence animal productivity and welfare. Environmental and genetic factors are critical for the development of these phenomena that are common in both dairy and beef cattle (72). A study using quantitative trait loci mapping and genome-wide association identified several large genomic regions linked with these events in Holstein dairy cows. Stem-loop mir-1256 was identified as being involved in the post-transcriptional regulation of gene expression associated with maternal calving difficulties (73).

Research on the expression of miRNAs related to genetic selection in livestock is still in its infancy; however, the above reports demonstrated that the expression of these molecules in cattle can be affected by age and genetic selection. Further work is needed to determine their functional implications and their potential for use in selective breeding programs aimed at improving animal health, welfare, and production performance.

### Management and Stress

Different management- and housing-related factors can promote the onset of stress in livestock and significantly affect physiological and productive parameters. Stress conditions alter miRNA biogenesis, the expression of mRNA targets, and the activities of miRNA-protein complexes (74). As a model of stress, Colitti et al. explored the revision of the social hierarchy group in cows focusing on milk exosome composition during the lactation period, and found that several miRNAs were DE (75). Functional pathway analysis of potential target genes of the DE miRNAs identified genes related to glucocorticoid receptor signaling and neurotrophic factor-mediated TRK receptor signaling. The neurotrophic signaling pathway is involved in the adaptive stress response and can be considered an alternative to glucocorticoid signaling in modulating the expression of corticotrophin-releasing hormone (CRH) (76). Based on the responsiveness to environmental stimuli and involvement in the signaling pathways described above, the authors identified miR-142a, miR-135, miR-320a, and miR-30-5p as potential markers of a mild stress response in dairy cows (75).

### Environmental Stress

High temperature is one of the main environmental factors affecting livestock production, welfare, and health. Cooling methods (e.g., shade, fans, and sprinklers) are used in dairy and feedlot farms to partially mitigate the negative effects of heat stress. MiRNAs play an important role in the transcriptional regulation of genes coding for proteins involved in heat stress response-related mechanisms (77, 78). Heat stress negatively influences dairy cattle homeostasis. Moreover, the chronic stress response is characterized by physiological as well as biochemical parameters that negatively affect, among others parameters, milk composition and mammary gland health through decreasing the immune response and increasing susceptibility to mastitis (79). In particular, heat stress seems to affect lipolysis and lipogenesis-related enzymes in bovine adipocytes (80). Given that mammary gland adipocytes can regulate the growth and biological function of the mammary epithelium, the authors (78) investigated the role of miRNAs in the mammary tissue of dairy cattle in response to different thermal conditions. Sequencing analysis and RT-qPCR were performed to identify miRNAs that were DE in the bovine mammary gland during heat stress. Seven miRNAs (downregulated: miR-21-5p, miR-99a-5p, miR-146b; upregulated: miR-145, miR-2285t, miR-133a, miR-29c) potentially targeting cellular and developmental processes, cell death, and the biosynthesis of secondary metabolites were found to be DE after heat stress (78). Significant changes in miRNA expression were also detected in a bovine mammary epithelial cell line (MAC-T) in response to heat stress compared with thermal-neutral conditions (81). MiRNAs associated with cell-growth arrest and apoptosis (miR-34a, miR-92a, miR-99, and miR-184), oxidative stress (miR-141 and miR-200a), and fat synthesis (miR-27ab and miR-221) were reported to be upregulated (81).

Interestingly, based on the analysis of different matrices, such as serum and mammary gland cells, the upregulation of miR-27b (77, 81) and the downregulation of miR-146b (77, 78) were shared between studies conducted on different cattle breeds exposed to heat stress.

Single nucleotide polymorphisms (SNPs) or mutations in the 3'UTRs of genes can affect protein expression by creating illegitimate binding sites for miRNAs (82). A novel SNP and a new binding site for miR-484 in the heat shock transcription factor 1 (*HSF1*) gene were identified in Chinese Holstein dairy cattle, suggesting that this miRNA has a role in the heat stress response (83).

Dairy breeds are typically more sensitive to heat stress owing to a high metabolic rate and higher-producing; however, thermal stress negatively affects food intake and, consequently, skeletal muscle growth. Under heat stress, miR-1246 was found to be significantly upregulated in the plasma of both dairy cows and beef cattle (77, 84). MiR-1246 directly targets poly(rC) binding protein 2 (*PCBP2*) and cAMP response element binding protein-like 2 (*CREBL2*), lung cell growth and apoptosis regulators, and is closely related to genes involved in innate immunity. Based on this evidence, Zheng et al. and Hu et al. proposed miR-1246 as a negative regulator of heat stress in bovine species (77, 84).

The results of recent studies have indicated that the elucidation of the miRNA-mediated stress regulatory network may provide new tools for the genetic improvement of heat tolerance in cattle. Selecting dairy cattle based on thermoresilience using miRNAs as biomarkers in mammary gland tissue or the bloodstream could be a valid future research avenue.

High-altitude hypoxia represents a stressful stimulus and affects the immune system of animals, decreasing cytokine release and altering immunoglobulin secretion (85). These events are consistent with chronic stress affecting the physical conditions and increasing the vulnerability of animals to infection and disease. Several studies have investigated the adaptation of cattle managed on pasture in high-altitude regions (86, 87). Kong et al. investigated the miRNA expression profiles of two breeds of cattle, and found that the levels of circulating miR-155 and miR-17-5p were upregulated in Jersey cows (86), whereas miR-let7a-5p, miR-19a, and miR-181a were upregulated in Holstein cows (87). In both studies, hypoxia induced the upregulation of miRNAs involved in acute phase response (APR) signaling (86, 87), which is known to promote homeostatic mechanisms in response to inflammatory stimuli (88). These miRNAs likely modulate inflammation-stimulating factors and the immune response, exerting an important role in the bovine resistance to high-altitude hypoxia, and represent potential candidate stress-related biomarkers.

## miRNAs Related to Health and Welfare in Small Ruminants

**Figure 2:**
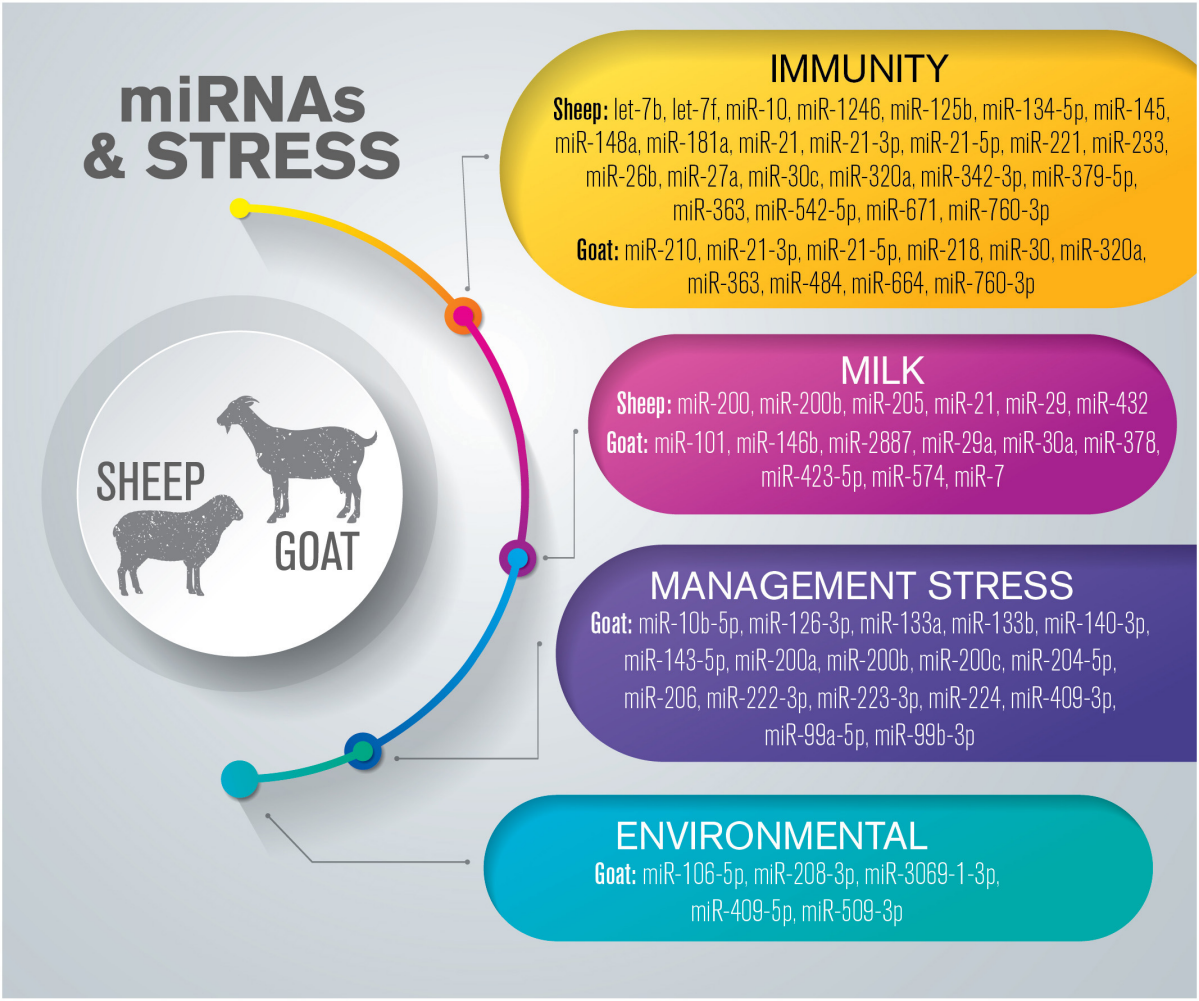
Overview of miRNAs dysregulated in response to stress in small ruminants. The list is limited to miRNAs confirmed by sequencing and validated through molecular approaches such as RT-qPCR and *in situ* hybridization.

Several hundred miRNAs have been identified in small ruminant species; however, the functional roles of many of these miRNAs in the various regulatory systems remain unclear. Studies in sheep and goats have often focused on economic traits such as milk, meat, and wool production (82, 89–92), and relatively few studies have investigated their health status related to welfare conditions (Figure 2 and Supplementary Table 2).

### Immunity

C-miRNAs have been proposed as biomarkers in the interaction between the HPA axis and stress-activated immune responses (93). A strong stress response in sheep was correlated with distinct circulatory biomarker profiles, demonstrating that stress resiliency may be determined by specific circulatory patterns. The inflammatory response, detected through the measurement of immune and stress biomarkers, was associated with high expression levels of miR-145, miR-233, and miR-1246 (93).

miRNAs have also been shown to modulate the immune response to infectious diseases in small ruminants. Peste des petits ruminants virus (PPRV) is a member of the Morbillivirus family, and is highly contagious and fatal for small ruminants, both domestic and wild (94). Qi et al. (95) demonstrated that the downregulation of miR-218 directly targeted signaling lymphocyte activation molecule (SLAM or *CD150*), which promotes the expression of other genes such as *IFNG, TNF*, and *IL10* during PPRV infection (95). PPRV has been shown to modulate the expression of at least 10 miRNAs in peripheral blood mononuclear cells (miR-664, miR-2311, miR-2897, miR-484, miR-2440, miR-3533, miR-574, miR-210, miR-21-5p, and miR-30). Of these, miR-664 and miR-484 were upregulated and were shown to exert proviral and antiviral activity, respectively. In contrast, miR-21-5p, which decreases the sensitivity of cells to the antiviral activity of interferons (IFNs), and miR-30b, which inhibits antigen processing and presentation by primary macrophages, were found to be downregulated in PPRV infection (96). Moreover, the authors showed that *TGFBR2* was directly targeted by both miR-21-5p and miR-484 (96). The influence of PPRV on miRNA expression levels was investigated in the spleen and lung of infected sheep and goats (97). Comparing the two species, 20 and 11 DE-miRNAs were shared in the spleen and lung, respectively. Gene ontology analysis for 6 DE-miRNAs (miR-21-3p, miR-1246, miR-27a-5p, miR-760-3p, miR-320a, and miR-363) indicated that these miRNAs were involved in several immune response signaling pathways, suggesting that PPRV-induced miR-21-3p, miR-320a, and miR-363 expression might act cooperatively to enhance viral pathogenesis (97).

A study investigating the ability of the Maedi–Visna virus (MVV) to modulate the response of the ovine lung at the molecular level demonstrated that several miRNAs were DE between clinically affected and seronegative sheep (98). Three DE-miRNAs, namely, miR-21, miR-148a, and let-7f, may have potential implications for host–virus interaction. In particular, miR-21, a regulator of inflammation and proliferation, may be a marker for lung lesion severity and/or a putative target for therapeutic intervention (98).

The profiles of exosomal miRNAs isolated from sheep pox virus (SPPV)-infected ovine testicular cells were characterized by He and colleagues (99), and 34 known miRNAs were found to be DE between infected and control cells. The levels of miR-21, miR-10b, and let-7f increased post-infection, whereas those of let-7b and miR-221 decreased. The candidate target genes were mostly involved in immune system processes and stimulus responses (99). Although the authors did not analyze the feasibility of using these miRNAs as potential biomarkers, further studies may cover this gap. Using the same ovine primary testicular cell culture, 25 known and 240 novel candidate miRNAs were shown to be DE following bluetongue virus (BTV) infection (100). Using RT-qPCR, the authors confirmed that let-7d, miR-29b, miR-29, and miR-61 were upregulated, whereas let-7f, mir-10b, miR-369-5p, miR-158, and miR-805 were downregulated. The ability of these miRNAs to modulate some of their target genes were also demonstrated through the measurement of target gene mRNA expression levels. The target genes were mainly involved in immune system responses and biological regulatory processes. The signaling pathways included the MAPK, PI3K/AKT, endocytosis, Hippo, NF-kB, FoxO, JAK/STAT, and TLR signaling pathways (100).

Transmissible spongiform encephalopathies (TSEs), also known as prion diseases, are fatal neurodegenerative disorders affecting humans, cattle, sheep, and goats (101). In 2017, Sanz Rubio and colleagues demonstrated that c-miR-342-3p and c-miR-21-5p levels were increased in the blood of sheep naturally infected with classical scrapie, demonstrating that these two miRNAs may be feasible biomarkers for these pathologies (102).

Parasites can also modulate the expression of miRNAs involved in inflammatory and immune responses. Two groups of sheep, resistant and non-resistant to cystic echinococcosis, were orally infected with *Echinococcus granulosus* eggs, which cause a chronic parasitic zoonosis, and their intestinal miRNome was compared (103). Eighty-three known miRNAs were significantly DE, 75 of which were upregulated and 8 downregulated. Moreover, six of them (miR-21-3p, miR-542-5p, miR-671, miR-134-5p, miR-26b, and miR-27a) showed significantly higher expression in resistant sheep compared with that in non-resistant animals. The potential target genes of these miRNAs were associated with inflammation and immune responses (103). Although no further investigation of these miRNAs has been performed to date, future studies should focus on evaluating their levels in body fluids, such as blood and saliva, to assess their potential as biomarkers for cystic echinococcosis, an important health problem for both animals and humans.

### miRNAs and Milk

Studies have identified miRNAs involved in mammary gland development in different phases of pregnancy (104), colostrogenesis, and lactogenesis in small ruminant species (105, 106). Galio et al. investigated the expression profiles of miRNAs in the mammary gland during pregnancy and lactation, and found that 100 were regulated according to three main expression patterns (104). At the beginning of pregnancy, when proliferation is most active, miR-21 was strongly expressed in epithelial cells of the normal mammary gland, while miR-205 was abundantly found in basal cells. During the second half of pregnancy, miR-205 expression was increased in luminal cells. The authors suggested that miR-205 may control the progenitor cell stock in cooperation with miR-200 to maintain epithelial status by repressing an epithelial–mesenchymal transition-like program and preserving the secretory phenotype of mammary epithelial cells. The expression of both miRNAs increased at the end of pregnancy and lactation playing an important role on mammary gland development (104).

MiRNA expression profiles during milk lactation and in the dry period have been reported for dairy goats. MiR-423-5p, miR-378, and miR-7 were identified as exerting important regulatory functions in milk ingredient transport and ingredient synthesis during lactation (105). Hou et al. compared the miRNA expression profiles of colostrum and milk collected during peak lactation, and reported that miR-574 may be involved in the development of the mammary gland and milk secretion in goats via the estrogen, endocrine, adipocytokine, oxytocin, and MAPK signaling pathways (106).

Another type of non-coding RNA, circular RNA (circRNA), exerts important roles in the ovine mammary gland. The most important function is to act as a “sponge” for miRNAs in cells by enriching miRNA binding sites (107). Through this mechanism, circRNAs can increase the expression levels of their target genes by abolishing the inhibitory effect of miRNAs. Eight DE-circRNAs with high expression levels and their corresponding target miRNAs and mRNAs, which create a miRNA–circRNA–mRNA network, were screened, demonstrating that the interaction between some key circRNAs and their target miRNAs (miR-432, miR-200b, and miR-29) are related to mammary gland development and milk secretion (108).

The dairy goat mammary gland and the produced raw milk shared 13 miRNAs among the most abundant members, suggesting that these miRNAs present in the milk had originated from the cells of the mammary gland (109). Furthermore, 4 of the 20 most abundant miRNAs (miR-29a, miR-30a, miR-101, and miR-146b) are immune-related miRNAs that target genes involved in immune responses, such as *IFNG* and genes involved in NF-κB and ICOS signaling. Most miRNAs, especially immune-related miRNAs, were expressed at similar levels in the mammary gland and milk, and the authors hypothesized that there was a correlation with the immature immune system requirements of the offspring (109).

### Animal Management-Related Stress

Weaning stress in young ruminants is induced by an inability to cope with a new diet, and is characterized by growth stasis (110). In goat kids, blood miRNA and mRNA signatures were investigated 1 day before weaning (44 days of age) and 3 days after weaning (48 days of age). Eighteen miRNAs (8 upregulated and 10 downregulated) and 373 genes were found to be DE (111). Two miRNAs related to skeletal muscle development (miR-206 and miR-133a/b) were significantly downregulated in post-weaning animals, as were cell proliferation-associated miRNAs (miR-99b-3p, miR-224, miR-143-5p, and miR-10b-5p). This suggested that weaning stress induced growth retardation in the animals through the repression of cell proliferation, and the authors proposed that significantly altered levels of these miRNAs may be potential biomarkers to assess the severity of weaning stress in goat production (111).

The nutritional status of animals is fundamental for their reproductive performance (112). Yang et al. treated ewes with or without concentrate added to the diet, and compared the levels of body condition score, estrus rate, and hypothalamus–ovary–nutrition-related miRNAs (113). A large number of DE-miRNAs were identified, including 148 that were expressed in the hypothalamus and 113 that were expressed in the ovary. Among the significantly DE-miRNAs, miR-200a, miR-200b, and miR-200c were expressed in both organs. Additionally, of the target genes predicted to be associated with nutritional status and seasonal reproduction in sheep, *GNAQ* was validated as a target of miR-200b. Furthermore, the authors demonstrated that miR-200b was involved in the regulation of estrus-related genes (*ITPR, PRKCB, GPR54*, and *KISS1*) in the hypothalamus–pituitary–ovary axis through the direct negative regulation of *GNAQ* in the hypothalamus (113). The effect of feed deprivation was investigated by Mobuchon et al. (114) who compared the miRNomes of the mammary glands of *ad libitum*-fed or food-deprived lactating goats. Seven highly expressed miRNAs (miR-99a-5p, miR-126-3p, miR-140-3p, miR-222-3p, miR-223-3p, miR-204-5p, and miR-409-3p) were validated as being dysregulated, while their potential target genes were associated with lipid metabolism (114). The authors highlighted that the expression of miR-204-5p and miR-223-3p was the most markedly affected by food deprivation, and that these miRNAs may target several genes with roles in the nutritional regulation of gene expression in the mammary gland (114).

### Environmental Stress

Very recently, some miRNAs were found to be DE under high-altitude hypoxia in both goats and cattle (115). Comparative miRNA transcriptome analysis of two goat populations at distinct altitudes produced reliable evidence for acclimatization based on miRNA–mRNA interactions in hypoxia-related pathways, including that associated with hypoxia inducible factor 1 (HIF-1). The downregulation of miR-509-3p, miR-3069-1-3p, miR-409-5p, and miR-208-3p, and the upregulation of miR-106a-5p, were reported in the hypoxia-sensitive tissues selected by the authors, including the heart, kidney, liver, muscle, and spleen, confirming that miRNAs have a regulatory role in mechanisms related to high-altitude adaptation (115). The potential target genes of these DE-miRNAs are involved in apoptosis, angiogenesis, DNA damage repair, erythropoiesis, and energy metabolism (115). Moreover, the authors demonstrated that miR-106a-5p exerted a negative regulatory effect on angiogenesis by targeting the *VEGFR1* (or *FLT1*) gene, thereby paving the way for further utilization of molecular resources in plateau regions.

## miRNAs Related to Health and Stress in Pigs

**Figure 3:**
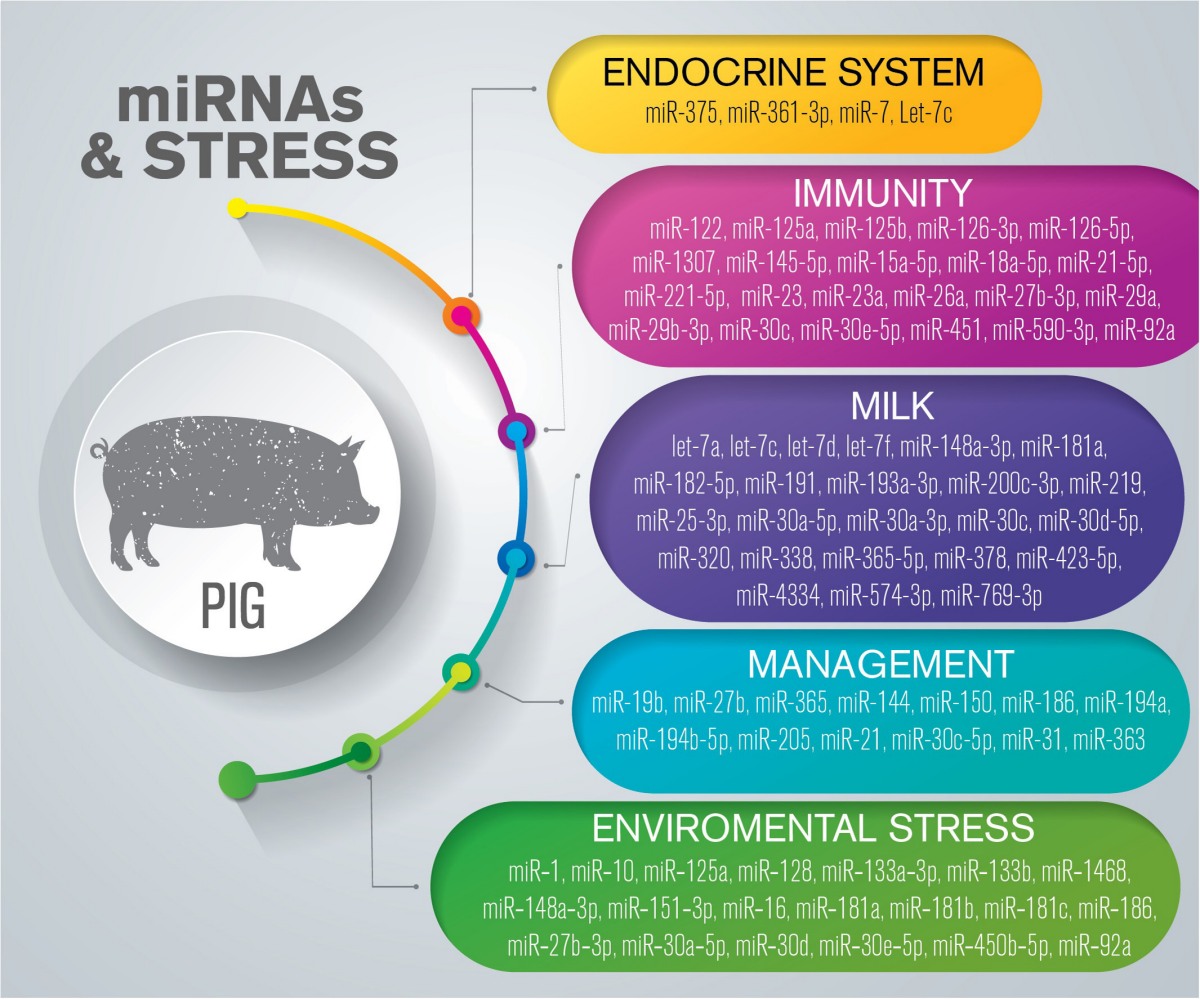
Overview of miRNAs dysregulated in response to stress in pigs. The list is limited to miRNAs confirmed by sequencing and validated through molecular approaches such as RT-qPCR and *in situ* hybridization.

Many of the effects of stressors are readily apparent on-farm, including irregular estrus expression, increased abortion rates, depressed offspring, and an increase in infection-associated pathologies. The effects of stress, imprinted in the life cycle of pigs by intensive breeding systems, involve changes in physiological pathways that affect the health and welfare of this species (Figure 3) (Supplementary Table 3).

### The Endocrine System and Response to Stress

The first reaction to a stressor is an adaptive HPA alteration. However, persistent stress can induce changes in gene regulatory networks, including alterations in miRNA levels (116). Recent studies have highlighted the roles of miRNAs in the regulation of the HPA axis in pigs (117–120).

During a stressful event, CRH activates the HPA axis, promoting the secretion of adrenocorticotropic hormone (ACTH) by the pituitary gland and glucocorticoids by the adrenal glands. The relationship between CRH and miR-375 in the regulation of catecholamine biosynthesis was investigated in the adrenal gland of female pigs, with the results demonstrating that CRH negatively regulated the expression of miR-375, which, in turn, negatively regulated the expression of catecholamines (117). MiR-375 directly targeted the *SP1* gene, a downstream effector of the protein kinase A pathway, decreasing steroidogenesis and the production of glucocorticoids (117).

Ye et al. investigated the ability of miRNAs to post-transcriptionally regulate the expression of gonadotropin follicle stimulating hormone (FSH) in porcine anterior pituitary cells *in vitro* (118), and demonstrated that the expression levels of miR-361-3p and the abundance of FSHB were negatively correlated. Following cell stimulation with gonadotropin-releasing hormone, the level of miR-361-3p decreased, whereas that of FSHB increased. The underlying mechanism was reported to be related to the ability of miR-361-3p to directly bind the 3′-UTR of *FSHB* (118). The ability of miRNAs to modulate FSH expression has also been reported in the context of zearalenone (ZEA), a non-steroidal *Fusarium* mycotoxin found in animal feed that can damage the reproductive system of animals (119). The effects of ZEA on the pituitary gland included the modulation of miR-7, which acts as a regulator of gonadotropin synthesis and secretion. The authors showed that ZEA enhanced miR-7 expression, which, in turn, inhibited FSH expression by directly targeting the *FOS* gene (119). Qi et al. explored the changes in miRNA expression after growth hormone–releasing hormone (GHRH) and cortistatin challenge in porcine pituitary cells and identified 19 and 35 DE miRNAs, respectively. Functional tests demonstrated that let-7c modulated the expression of growth hormone 1 (*GH1*) and growth hormone releasing hormone receptor (*GHRHR*) by binding to the respective 3′UTRs and promoting a decrease in GH secretion (120).

### Immunity

Several studies have investigated the involvement of miRNAs in the swine immune response to viruses, bacteria, and parasites, while others have compared healthy pigs of different breeds using sequencing, microarray, and RT-qPCR approaches (Supplementary Table 4). The genes targeted by miRNAs identified in these studies using *in silico* target prediction or experimental validation are involved in immune-related pathways such as apoptosis, inhibition of viral replication, and viral recognition (121–126). In this section, we have focused on studies that undertook experimental validation of target genes and demonstrated the potential of the miRNAs as biomarkers or for use in antiviral therapy.

The role of miRNAs as orchestrators of the innate immune response elicited by the H1N2 influenza A virus (IAV) in lungs and leukocytes has been investigated in an *in vivo* porcine model (121, 122, 127). Brogaard et al. demonstrated that several miRNAs and immune-related genes were DE in the lungs and leukocytes of infected pigs, and that modulation occurred at different time points (121, 122). In the lungs, the authors negatively correlated the expression of some miRNAs and genes. MiR-29b-3p and miR-15a-5p were shown to play a role in apoptosis *via* the targeting of the *BCL2* and *MCL1* genes; miR21-5p was reported to modulate the expression of both pro- and anti-inflammatory cytokines; and miR-18a-5p and miR-590-3p targeted the eukaryotic translation initiation factor 2 alpha kinase 2 (*EIF2AK2*) gene that encodes an inhibitor of viral replication (122). In leukocytes, different miRNAs were DE at three time points after infection, and the analysis of miRNA-gene interactions demonstrated that they were involved in apoptosis and innate immune responses (121). In conclusion, the modulatory activities of miR-29b-3p and miR-21-5p are conserved between lungs and leukocytes and these miRNAs represent potential candidate targets for the modulation of the innate immune response in pigs infected with H1N2 influenza. The ability of miRNAs to modulate the immune response during PRRSV infection has been extensively investigated. MiR-27b-3p and miR-26a were identified as inhibiting the replication of the virus by targeting PRRSV non-structural protein 2 in alveolar macrophages (125) as well as factors involved in the IFN pathway, including the *MX1* and *IFI44/IFI44* genes, chemokines, cytokines, and complement factor in MARC-145 cells (128). MiR-29a was shown to regulate viral replication by directly binding to its genomic RNA and promoting PRRSV replication through the targeting of the 3'UTR of *AKT3* (123). The ability of miRNAs to modulate IFN activation during infection has also been largely studied. MiR-23 was reported to increase type I interferon (*INF-1)* expression by targeting *IRF3/IRF7*, which can further suppress PRRSV infection (129); meanwhile, miR-30c can be upregulated by PRRSV to impair IFN-I signaling by directly binding to the *JAK1* and interferon-alpha/beta receptor beta chain loci, and thereby promote viral replication (130).

The effect of African swine fever virus (ASFV) infection on pig miRNAs has been investigated *in vivo* by comparing miRNA expression in animals infected with two viral strains (virulent vs. attenuated) (131). Ten miRNAs were selected for target prediction based on the highest representation by tissue and conditions, namely, miR-23a, miR-30e-5p, miR-92a, miR-122, miR-125b, miR-126-5p, miR-145-5p, miR-125a, miR-451, and miR-126-3p. The putative target genes were related to immune responses, such as B and T cell receptor signaling pathways, natural killer cell-mediated cytotoxicity, or Fc gamma R-mediated phagocytosis, as well as with processes related to infection-related pathogenesis and virus–host interaction (131). In another *in vivo* study, the same authors assessed the potential for ASFV to encode its own miRNAs, with negative results (132).

Mir-1307 was reported to be upregulated in porcine kidney cells, pre-activating and enhancing signaling by the innate immune system at the early stage of foot-and-mouth disease virus (FMDV) infection. Mir-1307 promoted the degradation of the structural viral protein VP3 through the proteasome pathway and the upregulation of immune-related genes (126). The therapeutic potential of miR-1307 was also demonstrated by the subcutaneous injection of miR-1307 agomir into suckling mice, where it delayed FMDV-induced lethality (126). The upregulation of miR-221-5p in MARC-145 cells infected with porcine epidemic diarrhea virus (PEDV) resulted in the inhibition of viral replication through the direct targeting of the 3′UTR of PEDV and enhancing the host immune response (124).

### miRNAs and Milk

Milk exosomes can actively deliver their cargo, including miRNAs, from donor to recipient cells, thereby regulating target gene expression and recipient cell function, and can reach different tissues after intestinal absorption (133). Using a deep-sequencing approach, Gu et al. investigated the lactation-related miRNA expression profile in porcine milk exosomes across the entire lactation period (newborn to 28 days), focusing on the expression of immune-related miRNAs (134). They demonstrated that immune-related miRNAs (miR-148a-3p, miR-182-5p, miR-200c-3p, miR-25-3p, miR-30a-5p, miR-30d-5p, and miR-574-3p) were present in higher numbers in the colostrum than in the mature milk, and were also more abundant in the blood of colostrum-only fed piglets compared with that of milk-only fed piglets (134). MiRNAs delivered by swine milk exosomes were also characterized using sequencing, and the 10 most abundant miRNAs were identified as miR-193a-3p, miR-423-5p, miR-320, miR-181a, miR-30a-3p, miR-378, miR-191, let-7a, let-7f, and let-7c (135). MiR-30a and members of the let-7 family are shared between the two studies, highlighting their crucial role in safeguarding the health of the newborn. The effect of dietary ginseng polysaccharide (GPS) supplementation on milk miRNAs was investigated to evaluate the ability of GPS to modulate the piglet immune response. The miRNA exosome cargo purified from milk samples was sequenced, leading to the identification of 10 miRNAs that were upregulated, and 16 that were downregulated, in the GPS-treated group compared with the control group. These miRNAs were suggested to act as potential regulators of immune functions (136). The expression levels of miR-30a and let-7d were altered by GPS supplementation, again highlighting their essential role in milk. Xie et al. demonstrated the protective effect of porcine milk miRNAs by investigating the role of miR-181a, miR-30c, miR-365-5p, and miR-769-3p in milk exosomes. The high expression of these miRNAs was correlated with a significant decrease in the expression levels of their target genes and encoded proteins that are involved in the p53 pathway (137). Using the same IPEC-J2 model, the authors previously demonstrated that, after lipopolysaccharide (LPS) exposure, miR-4334, miR-219, and miR-338 targeted the *TLR4, MyD88*, and *TP53* genes, respectively, thereby reducing LPS-induced apoptosis through the TLR4/NF-κB and p53 pathways (137, 138).

### Management and Stress

Routine husbandry procedures in piglets, such as castration and tail docking, are acute pain-associated events that threaten animal welfare. In a very recent report, and for the first time in a swine species, the concentrations of salivary miRNAs were investigated and the expression levels of miR-19b, miR-27b-3p, miR-215, miR-22-3p, miR-155-5p, miR-365-5p, and miR-204 were assessed for their potential as pain biomarkers by RT-qPCR (24). The authors demonstrated that miRNAs were more abundant in animals where the pain was not mitigated by the use of an anesthetic, and proposed these miRNAs as potential biomarkers for pain identification in these events. In particular, miR-19b, miR-365, and miR-27b were correlated with the inflammation resulting from these common husbandry practices (24).

To elucidate the role of miRNAs during the stressful weaning transition, Tao and Xu (139) used high-throughput sequencing to compare the miRNA profiles of the jejunum and serum collected from piglets after weaning and during the suckling period. A large number of miRNAs were identified as being DE at days 1, 4, and 7, and many were found to be involved in the modulation of small intestinal metabolism, stress responses, and immune functions (139). Three c-miRNAs (miR-21, miR-31, and miR-205) were upregulated and 7 (miR-30c-5p, miR-144, miR-150, miR-186, miR-194a, miR-194b-5p, and miR-363) downregulated in weaned piglets compared with suckling piglets. As the expression of miR-194b-5p was significantly downregulated in the serum and small intestine of the weaned piglets, the authors hypothesized that its expression in serum may reflect the decreased expression in the small intestine. This finding is important given that miR-194b-5p has been demonstrated to downregulate the expression of small ubiquitin-like modifier 2 (*SUMO2*) (140). SUMO2 is pivotal for cell homeostasis during endogenous or environmental stress, including, among others, heat shock and nutrient stress (141). Although the role of miR-194b-5p as a potential biomarker has yet to be investigated, it may represent a candidate biomarker to monitor the intestinal health of piglets during the weaning period.

### Environmental Stress

Because pigs lack sweat glands and have relatively small lungs when compared with their body mass, they are more sensitive to heat than other livestock species (142). Furthermore, new pig genetic lines produce nearly 20% more heat than previous breeds (143). Heat stress affects several aspects of pig farming, including reproductive performance, feed intake, body condition, immune response, and milk production (144–148). Heat stress also influences muscle development, modifying the balance between protein synthesis and degradation (149). Several studies have investigated the role of miRNAs in muscle development under different environmental conditions and in different breeds (150–154). Hao et al. investigated for the first time how heat stress affected the muscle miRNA expression profile of cross-bred barrows (155). Castrated pigs were raised under either a constant environmental temperature of 22°C or a constant high environmental temperature of 30°C for 21 days. Sequencing of samples collected from the *longissimus dorsi* muscle demonstrated that 58 miRNAs were DE, 30 of which were downregulated and 28 upregulated (155). Of the genes potentially targeted by the DE-miRNAs, the authors analyzed only those that were related to muscle metabolism and stress response, and found that the expression of pyruvate dehydrogenase kinase 4 (*PDK4*), heat shock protein 90 (*Hsp90*), desmin (*DES*), lactate dehydrogenase A (*LDHA*), and stearoyl-CoA desaturase (*SCD*) exhibited an inverse correlation with the DE-miRNAs (155).

Combined, these results demonstrated that heat stress can modulate the expression of miRNAs in the muscle and mammary gland and, accordingly, also that of downstream genes, influencing not only tissue structure but also function, glycolysis, and lactate and lipid metabolism. Consequently, both animal welfare and production performance can be affected, indicating that accurate identification and validation of miRNAs and their target genes are essential for the development of novel regulatory gene-based breeding strategies. Addressing this issue is of paramount importance for understanding, and potentially modulating, the capacity of both individuals and herds to adapt to climate change-related heat stress.

## miRNAs Related to Health and Stress in Poultry

**Figure 4:**
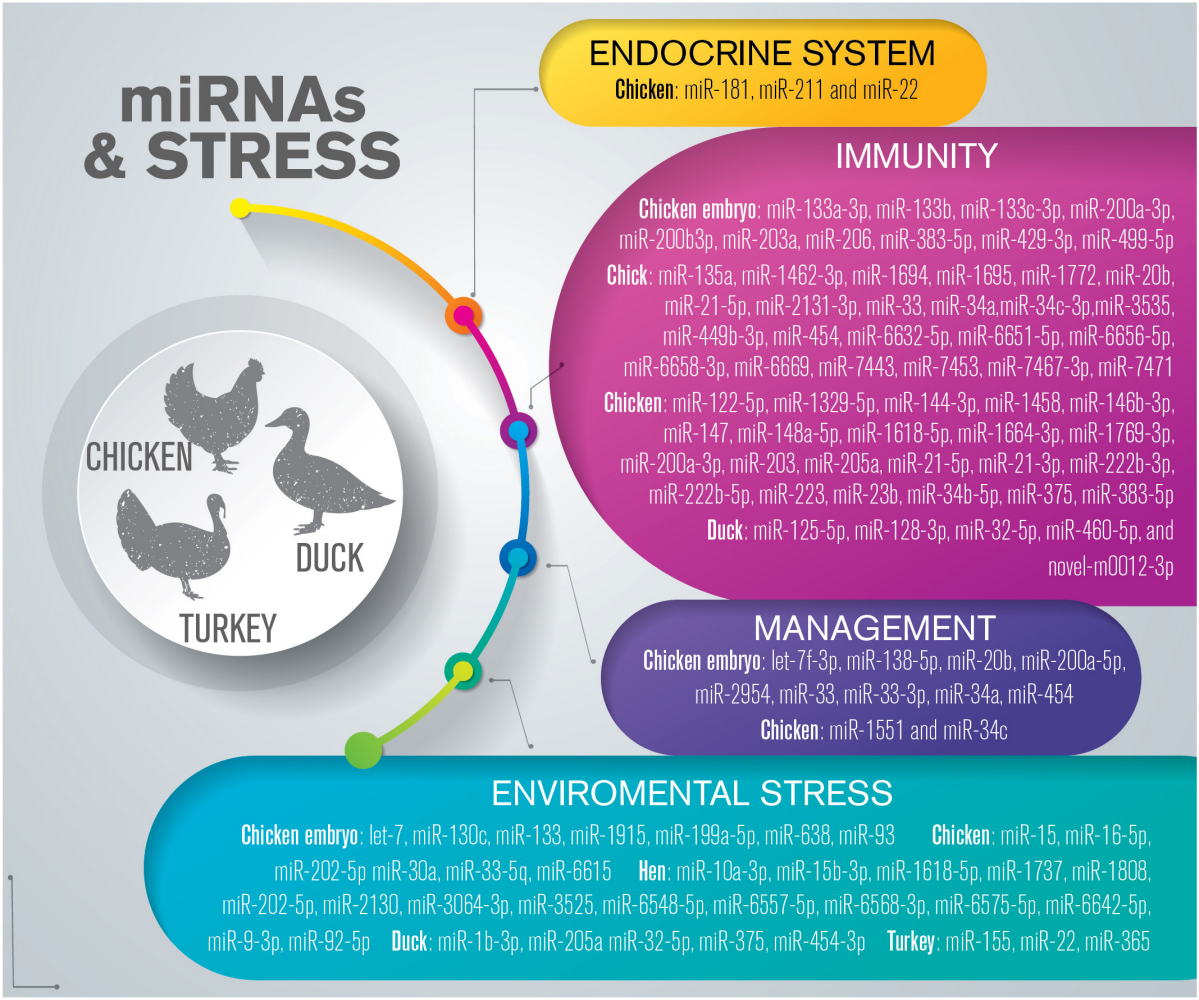
Overview of miRNAs dysregulated in response to stress in poultry. The list is limited to miRNAs confirmed by sequencing and validated through molecular approaches such as RT-qPCR and *in situ* hybridization.

Several stressors, including temperature, stocking density, restraint, cooping, shackling, fasting, feed restriction, dietary protein deficiency, and fear, can induce stress in poultry, triggering an increase in circulating concentrations of the adrenocortical hormone, corticosterone, and the heterophil: lymphocyte ratio, although whether corticosterone is a reliable indicator of stress remains inconclusive (156). Recent studies have shown that miRNAs are involved in the regulation of health and stress-related events in poultry species (Figure 4) (Supplementary Table 5).

### The Endocrine System and Its Response to Stress

The role played by miRNAs in the modulation of fear- and stress-related genes in broiler chickens was investigated for the first time in 2014 (157). Using a model of tonic immobility, Wang and colleagues identified three miRNAs—miR-181, miR-211, and miR-22—predicted to target the glucocorticoid receptor in the chicken hypothalamus by comparing the hypothalamic expression of genes in the serotonergic system and the HPA axis under basal and corticosterone-exposed conditions (157).

### Immunity

Host–pathogen interactions are critical for understanding infectious disease-related pathogenesis and immune responses. The identification of dysregulated miRNAs resulting from host–virus cross-talk in poultry (chicken and ducks) has recently been summarized by Duan et al. (42), who reviewed the literature reporting on the modulation of miRNAs during Marek's disease virus (MDV), avian leukosis virus subgroup J (ALV-J), IAV, infectious bursal disease virus (IBDV), infectious bronchitis virus (IBV), and reticuloendotheliosis virus strain T (Rev-T) infections. Focusing on the ability of miRNAs to coordinate the poultry immune response, several authors recently identified DE-miRNAs in different tissues (spleen, lung, and kidney) of chicken embryos infected with IBV and IBDV using deep-sequencing approaches (158–160). Different viral infections seem to induce the dysregulation of the same miRNAs, such as miR-203a, miR-200a-3p, miR-200b3p, miR-429-3p, miR-133c-3p, miR-133a-3p, miR-133b, miR-206, and miR-499-5p, indicating their crucial role in the host response in poultry. Nuclear factor of activated T cells 3 (*NFATC3*), *NFAT5*, signal peptide peptidase like 3 (*SPPL3*), and transforming growth factor beta 2 (*TGFB2*), known immune-related genes, were negatively correlated with the upregulated DE-miRNAs identified by the same authors. IBV can also influence the activities of bone marrow-derived dendritic cells by modulating the expression of 19 miRNAs (upregulated: miR-135a, miR-7471, miR-7453, miR-7443, miR-1695, miR-1772, and miR-6669; downregulated: miR-6632-5p, miR-7467-3p, miR-449b-3p, miR-6658-3p, miR-2131-3p, miR-34c-3p, miR-1694, miR-3535, miR-21-5p, miR-1462-3p, miR-6656-5p, and miR-6651-5p) (160).

ALV-J causes immunosuppression and damage in poultry immune organs, including the spleen, bursa, and thymus (161). Lan et al. (162) investigated the molecular network in the spleen after ALV-J infection, and showed that, at 40 days post-infection, 864 genes, 7 miRNAs (miR-205a, miR-21-5p, miR-21-3p, miR-383-5p, miR-203, miR-223, and miR-148a-5p) and 17 long non-coding RNAs were DE. The combined analysis of long non-coding RNA and miRNA networks highlighted their relationship with several DE-genes associated with the immune response (162). The expression levels of miR-21-5p, miR-21-3p, miR-223, and miR-148a-5p were also dysregulated in bone marrow-derived dendritic cells after ALV-J infection, demonstrating the importance of this miRNA panel in the regulation of poultry immune responses (130). To promote its survival, ALV-J induces the expression of miR-34b-5p and miR-23b in the chicken spleen, leading to the downregulation of melanoma differentiation-associated gene 5 (*MDA5*) and interferon regulatory factor 1 (*IRF1*), respectively, genes that are involved in the IFN pathway (163, 164).

The poultry immune system is also activated by REV, leading to differential miRNA expression in the host. Gao et al. identified 27, 29, and 29 DE-miRNAs in the spleen of *in vivo* REV*-* infected chickens at 7, 14, and 21 days post-infection, respectively (165). The authors demonstrated a negative correlation between 14 miRNAs (miR-200a-3p, miR-375, miR-1458, miR-1664-3p, miR-122-5p, miR-222b-5p, miR-147, miR-1329-5p, miR-1618-5p, miR-1664-3p, miR-146b-3p, miR-222b-3p, miR-144-3p, and miR-1769-3) and 14 target genes, all of which were involved in a variety of biological pathways, including the endocrine system, the immune system, cell growth, and cell death (165).

To elucidate the immune-related mechanisms activated in response to duck hepatitis A virus type 3 (DHAV-3) infection, the miRNA and mRNA expression profiles of duckling liver tissues infected with lethal DHAV-3 were determined through sequencing (166). The analysis showed that miR-32-5p, miR-125-5p, miR-128-3p, miR-460-5p, and novel-m0012-3p are potential regulators of immune-related signaling pathways, including cytokine–cytokine receptor interaction, apoptosis, TLR, FoxO, and JAK/STAT signaling pathways during DHAV-3 infection (166).

### Management-Related Stress

Feed supplementation and feed deprivation, combined with the genetic variation acquired by poultry breeds through artificial selection, can affect the plasma or tissue levels of stress-responsive miRNAs (167–171). By developing specific feed supplementation and nutrition plans, it is possible to define strategies to promote poultry welfare and health, while preserving competitive production levels.

Several studies have investigated the effects of mineral supplementation in poultry farming (168, 169). Feed deprivation for 48 h was employed to evaluate the modulation of miRNA expression and its effect on hepatic metabolic pathways (170). The levels of miR-33, miR-20b, miR-34a, and miR-454 were affected by delaying feed consumption, which, in turn, influenced the expression of target mRNAs such as *FADS1*, encoding an enzyme involved in fatty acid synthesis, and *FOXO3*, which encodes a transcription factor known to protect cells against oxidative stress. Both *FADS1*and *FOXO3* are targeted by miR-20b (170). Selenium deprivation can also modulate the expression of miRNAs in the chicken. Different reports have described selenium deficiency as promoting tissue damage, including cell apoptosis, through triggering oxidative stress and the dysregulation of miRNA expression (170–174). In broiler cardiomyocytes, this deprivation promotes the upregulation of miR-200a-5p, which triggers myocardial necroptosis by targeting ring finger protein 11 (*RFP11*) (171), as well as the upregulation of miR-2954, leading to the autophagy and apoptosis of myocardial cells during cardiac injury through the PI3K pathway (172). Selenium deficiency induces skeletal muscle injury through the induction of oxidative and endoplasmic reticulum stress. This effect is exerted *via* the upregulation of let-7f-3p expression, which downregulates the expression of selenoprotein K, a protein responsible for maintaining the physiological function of skeletal muscle (173). In chicken chondrocytes, the level of miR-138-5p increases after selenium deprivation, which promotes the overexpression of genes involved in mitochondrial apoptosis, including caspases 3 and 9, *BAX*, and *BAK*, as well as that of oxidative stress-related genes, such as selenoprotein M (174). In the broiler, selenium deprivation promotes endothelial cell apoptosis by decreasing the levels of miR-33-3p (175). Supplementing poultry diet with selenium might prevent miRNA-mediated pathological muscle injury, including apoptosis and vascular disease, thereby improving the health status of the animals and the nutritional quality of their meat products.

The effect of maternal manganese supplementation during thermal stress on the miRNA expression in broilers has also been investigated (176). The levels of miR-1551 and miR-34c were reduced in the hearts of chicken embryos derived from mothers fed manganese-supplemented diets and exposed to high temperatures through targeting BCL2 and NF-kB, highlighting that manganese supplementation may protect offspring from thermal injury (176).

### Environmental Stress

Birds are homeothermic, i.e., they can maintain their body temperature through the use of plumage as a thermal buffer, fat insulation, and salt glands (177). Heat stress exerts a marked impact on poultry welfare, health, and production, while also affecting feed intake, growth rate, mortality, egg production, hatchability, and other important traits governing the economic success of the poultry industry (168, 178, 179).

Zhu et al. investigated the plasma mRNA and miRNA profiles of laying hens to identify heat stress-responsive miRNAs and explore the potential mechanism underlying the role of miRNA–mRNA interaction in the regulation of the heat stress response (180). Sixteen miRNAs were found to be DE, 11 of which were upregulated (miR-2130, miR-92-5p, miR-1618-5p, miR-3064-3p, miR-6575-5p, miR-1737, miR-3525, miR-10a-3p, miR-6557-5p, miR-6568-3p, and miR-6548-5p) and five were downregulated (miR-15b-3p, miR-1808, miR-202-5p, miR-9-3p, and miR-6642-5p). The authors compared the DE genes and the putative targets of the altered miRNAs and identified 82 candidate target genes with potential roles in cellular responses to stress and lipid metabolism (180). The response to thermal stress at the morphological and molecular levels were investigated in the intestine of ducks exposed to high (30–40°C) or control temperatures (25°C) (181). By comparing the same tissues under different conditions, they identified 16, 18, and 15 miRNAs that were dysregulated between the duodenum, jejunum, and ileum, respectively, of control and heat-treated ducks. Among these miRNAs, miR-205a was dysregulated in both the duodenum and jejunum, miR-32-5p and miR-375 were DE in the ileum and duodenum, and miR-1b-3p and miR-454-3p were DE in the ileum and jejunum. Bioinformatic analysis indicated that several DE-miRNAs may be potentially involved in high-temperature induced-injury in ducks (181). The ability of temperature to remodel cells and tissues has been explored in broiler embryos (182). After exposure to different temperatures, miRNAs involved in myogenesis and body size (upregulation of miR-133 in breast muscle and downregulation of miR-199a-5p, miR-1915, and miR-638 in hind muscle), and cell proliferation and differentiation (downregulation of let-7, miR-93, and miR-130c) were dysregulation at embryonic day 7–10 after heat stress (38.5°C) and at embryonic day 10–13 after exposure to low temperature (36.5°C) (182). These observations suggest that the levels of a large number of miRNA–mRNA pairs are altered in various tissues under different temperature-related experimental conditions *in vivo*. These changes could be a result of animal adaptation to environmental conditions such as heat stress. Changes in embryonic incubation temperatures can result in phenotypic variations in chickens (183). MiRNA regulatory networks may be important for long-term transcriptional changes in response to embryo thermal treatment, and may have a role in the resistance to post-natal thermal stress.

The effects of environmental pollutants, such as cadmium and lead, on miRNA regulation have recently been investigated in poultry. When exposed to cadmium, chickens display lower levels of miR-33-5q in the spleen, which in turn leads to the suppression of AKT/mTOR signaling and HSP70 activity, and the upregulation of NF-κB, p-JNK/JNK, and genes involved in autophagy (184). The same group demonstrated that cadmium can also impair signaling through the miR-30a/GRP78 axis, thereby increasing ER stress, activating the IRE-1/JNK pathway, and promoting autophagy in the chicken kidney (185). Yin and colleagues investigated the effect of environmental lead contamination on the immune response of chickens (186), and demonstrated that lead can induce neutrophil apoptosis by increasing the expression of miR-16-5p and thus modulating the expression of genes (*PiK3R1* and *IGF1R*) responsible for regulating apoptotic pathways and oxidative stress (186). Ammonia is one of the main pollutants in housing associated with intensive chicken farming, and is produced through the combination of protein consumption, litter, and manure (187). Ammonia can affect the expression of miR-6615, a modulator of inflammatory responses, via the NF-κB pathway in the broiler spleen, as well as that of miR-202-5p, which regulates autophagy through the PTEN/AKT/mTOR pathway in the chicken heart (188, 189). The same group demonstrated that ammonia upregulates miR-15a expression in splenic lymphocytes, which induces mitochondria-mediated inflammatory responses and apoptosis by targeting BCL2 (190).

Road transportation is an issue in poultry welfare management, representing one of the most stressful events in the life of a turkey (191). Lecchi et al. (192) demonstrated that the serum levels of three c-miRNAs (miR-22, miR-155, and miR-365) were altered in turkeys after transportation, and may have diagnostic value in discriminating between stressed and unstressed animals.

## Conclusions and Future Perspectives

For consumer perception and product acceptance, as well as for the livestock industry, enhancing animal health and welfare are pivotal for improving the quality of food products. Within this context, objective and quantifiable laboratory markers that are easy to collect and conducive to defining the life quality and well-being of an animal are still lacking. The studies carried out to date have largely focused on stress or resiliency biomarkers, and many of these have focused on miRNAs that can provide information on the regulation of different processes across the tissue–biofluid channel. Conventional biomarkers may be useful as markers for stress and injury, but they provide limited information about the cellular mechanism underlying animal adaptation to adverse events. Extracellular miRNAs can be combined with other phenotypic measurements to more accurately monitor the stress responses of individual animals or populations, allowing producers to monitor changes in animal husbandry or production systems and determine whether these changes can reduce or eliminate the physiological effects of stress.

Here, we have reviewed the role of miRNAs in livestock stress induced by management conditions (e.g., housing, road transport), environmental challenges (including thermal stress), and disease, as well as the implications for immune responses and productive performance.

miRNAs have a leading role in all pathophysiological processes and some may be useful as potential biomarkers. Importantly, stressor-associated miRNA biomarkers can be detected in biological matrices such as milk, saliva, tears, cerebrospinal fluid, urine, and hair. If combined with routine blood collection, these advantages allow for less invasive monitoring of the health of an animal and/or the prevention of pathologies (26, 193). However, several challenges associated with the detection of c-miRNAs remain. The lack of a standard protocol for the quantification of c-miRNAs limits the comparison of miRNA expression profiles among different laboratories. The recognition of common standardized methods to minimize possible bias and raw data normalization also remains a critical issue. To establish c-miRNAs as novel biomarkers, their cellular origins and the relationship with their tissues must be clarified, as must their relationship with stressors. In a second step, large-scale studies are required to compare the expression of miRNAs across livestock species under stressful conditions of variable duration and strength so as to validate the applicability of miRNAs as biomarkers.

In cows and pigs, several miRNAs with potential as biomarkers for the management and regulation of stress and the control of animal health have already been identified. Although this information is still lacking for other livestock species, such as small ruminants and poultry, this gap will likely be bridged owing to the increase in our knowledge of miRNAs. Despite research on livestock miRNAs lagging behind that of humans, livestock miRNAs show great potential as a new class of stress- and health-related biomarkers and represent a new and exciting field to assess the management and welfare of animals.

## Author Contributions

SM and MB conceived the paper. SM, CL, FC, and MB wrote the manuscript. All authors revised and approved the final manuscript.

## Conflict of Interest

The authors declare that the research was conducted in the absence of any commercial or financial relationships that could be construed as a potential conflict of interest.
