# Supplementary material for: MicroRNAs as Biomarkers for Animal Health and Welfare in Livestock
Source: Front Vet Sci. 2020 Dec 18;7:578193. doi: 10.3389/fvets.2020.578193 (PMC7775535; doi:10.3389/fvets.2020.578193)
Supplement: Supplementary file 1 [file Table_1.pdf]

Supplementary Material

Table 1_Putative biomarkers and DE-miRNAs for experienced stress or stress susceptibility in bovine specie

	Model/disease	Target organ-tissues	DE-miRNAs Modulation	Predicted target	Reference
Immunity	*Mycobacterium bovis*	Ileal tissue<br>milk	miR-19, miR-196, miR-146<br>N/A modulation	Gene involved in immune and inflammatory responses	(50) (51)
	Streptococcus uberis-mastitis	Mammary gland	↓miR-181a, miR-16, miR-31<br>↑ miR-223	Genes involved in immune (Toll-like receptor - TLR-), metabolic, and cell growth-related signaling pathways	(56)
		Mammary gland<br>Blood (CD14+ monocytes)	↓miR-149, miR-146<br>↑miR-223	TLR, NOD-like receptor (NOD), and the RIG-I-like receptor (RGI-I) signaling pathways	(57)
	Staphylococcus aureus-mastitis	Mammary gland	↑ miR-223, miR-21-3p	Immuno-regulatory functions on innate immune-related genes (e.g. CXCL14 and KIT)	(58)
		Mammary gland	↓miR-26a, miR-26b miR-2373, miR-423-3p, miR-126, miR-19b, miR-148a, miR-21, miR-31, miR-143, miR-145, miR-2881, miR-200b, miR-99a, miR-30a-5p	Genes involved in immunity, inflammation, cell proliferation and apoptosis, tissue damage and repair. *FGA* gene (bridging molecule involved in host defense	(59)

Immunity			↑ miR-1343-5p, miR-2407, miR-296, miR-2360, miR-2374, miR-2328-3p, miR-2412, miR-2904, miR-494, miR-2392, miR-2898	mechanism)	
		Mammary gland	↓ miR-6522, miR-885<br><br>↑miR-7863**,** miR-874, miR-214, miR-382, miR-155	Gene involved in immune signaling pathways, including TLR signaling pathways, MAPK signaling pathway, cell adhesion molecules, TGF-β signaling pathway, leukocyte trans endothelial migration, cytokine-cytokine receptor interaction, and chemokine signaling pathways.	(52)
		Peripheral blood	↑ miR-144, miR-125	Genes involved in chemokine signaling pathway, mRNA surveillance pathway,neurotrophin signaling pathway, TGF-beta signaling pathway, MAPK signaling pathway and TLR signaling (TNF, CD14, MAPK12, MAPK3, MAPK14, TRAF6 and MAPK8)	(54)
	Escherichia coli-mastitis	Mammary gland	↓miR-415, miR-6516<br><br>↑miR-202, miR-2537, miR-1307, miR-326,	NR	(52)

			miR-378c, miR-432		
		Peripheral blood	↓ miR-342, miR-326, miR-331-3p<br><br>↑ miR-200, miR-205, miR-182, miR214, miR-145	Genes involved in innate immunity and inflammatory response: cytokine-cytokine receptor interaction, chemokine signaling pathway, leukocyte transendothelial migration, T cell receptor signaling pathway, TLR signaling pathway, and cell adhesion molecules	(60)
Colostrum and milk	Healthy	Colostrum	↑ miR-181, miR-155, miR-223	NR	(69)
			↓miR-222, miR-1248, miR-101, miR-21-5p<br><br>↑miR-142-3p, miR-29c, miR-340, let-7	Genes involved in milk synthesis (milk fat and protein metabolism) and immunity pathway PI3K-Akt signaling pathway, endocytosis, Ras signaling pathway, MAPK signaling pathway, and TNF signaling pathway	(67)
	mastitis	Milk	↑miR-146b, miR-222	Genes involved in inflammatory pathway	(62)
	Staphylococcus aureus-mastitis	Milk	↑miR-223, miR-142-5p	Genes involved in inflammatory, immune, and cancer pathways	(61)

Genetic background and age	Genetic-line related	plasma	miR-15a, miR-1256<br>N/A modulation	Genes involved in maternal calving difficulty	(71, 73)
	Age related	plasma	miR-27a-5p, miR-29a, miR-29c, miR-31, miR-126-5p, miR-192, miR-205, miR-215, miR-127, miR-140, miR-154c, miR-380-3p<br>N/A modulation	Expression changes occurring between calf, heifer and cow.<br>Target genes involved in body growth and tissue differentiation, lipid and protein metabolism.	(71)
Management stress	Social group revision	Milk exosome	↑ miR-142-3p, miR-142-5p, miR-135, miR-320a, miR-30-5p, miR-2284z, miR-146	Genes involved in neurotrophin-mediated cell survival, including RAS superfamily of small GTP-binding proteins (RAC1, RAP1A and RASA1),	(75)
Environmental stress	Heat	Serum	↓miR-26a, miR-146a, miR-146b, miR-199a-3p<br><br>↑miR-19a, miR-19b, miR-27b, miR-30a-5p, miR-181a, miR-181b, miR-345-3p, miR-1246	PCBP2 and CREBL2 genes involved in lung cell growth and apoptosis regulators. TLRsignaling pathway	(77, 84)
		Mammary gland	↓ miR-21-5p, miR-99a-5p, miR-146b<br><br>↑ miR-145, miR-2285t, miR-133a, miR-29c	Genes involved in regulation of Wnt, TGF-β, MAPK, Notch, and JAK-STAT pathways	(78)

		Mammary gland cell line (MAC-T)	↑miR-34a, miR-92a, miR-99, miR-184, miR-141, miR-200a miR-27a, miR-27b	Genes involved in apoptosis (downregulatedcyclin D2 and cyclin E2), oxidative stress and fat synthesis (downregulated FASN and PPARG genes)	(81)
		Blood	miR-484<br>N/A modulation	*HSF1* gene (direct target)	(83)
	High altitude hypoxia	plasma	↓miR-181a, miR-143<br><br>↑let7a-5p, miR-17-5p, miR-19a, miR-199-3a miR-155-5p	Genes involved in regulation of inflammatory homeostasis by inhibiting the acute phase response, coagulation system, and complement system and promoting LXR/RXR activation<br>↑ APR (Acute phase response) signaling, activating the LXR/RXR and FXE/RXR pathways	(86,87)

N/A= not applicable
