# Supplementary material for: MicroRNAs as Biomarkers for Animal Health and Welfare in Livestock
Source: Front Vet Sci. 2020 Dec 18;7:578193. doi: 10.3389/fvets.2020.578193 (PMC7775535; doi:10.3389/fvets.2020.578193)
Supplement: Supplementary file 2 [file Table_2.pdf]

Supplementary Material

Table 2_Putative biomarkers and DE-miRNAs for experienced stress or stress susceptibility in small ruminants species

	Specie	Model/disease	Target organ-tissues	DE-miRNAs Modulation	Predicted target	Reference
Immunity	*Ovis aries*	Endotoxemia by lipopolysaccharide (LPS)	Serum	↑ miR-145, miR-233, miR-1246	Genes involved in inflammatory response	(93)
		PPRV	Spleen	↑miR-2887-1, miR-2887-2, miR-17-3p, miR-486, miR-146b, miR-363, miR-451, miR-193a-3p, miR-760-3p, miR-144, miR-21-5p<br><br>↓miR-199b, miR-1271, miR-217, miR-6119-3p, miR-221, miR-744, miR-30c, let-7a-5p-2, miR-211	Genes involved in immune response and apoptosis signalling pathways	(98)
			Lung	↑ miR-320a-1, miR-320a-2, miR-1246, miR-363, miR-760-3p, miR-21-3p<br><br>↓miR-34b, miR-150	Genes involved in immune response and apoptosis signalling pathways	(97)
		VMV	Lung	↑ miR-21, miR-148a, let-7f, miR-379-5p<br><br>↓ miR-125b, miR-181a, miR-30c, let-7b	Genes involved in apoptosis, proliferation and growth, (PI3K-Akt and AMPK pathways)	(98)

		SPPV	Testis primary cells	↑ let-7f, miR-21, miR-10<br><br>↓ let-7b, miR-221	Genes involved in immune system processed and stimulus responses (MAPK signaling pathway, autophagy, Hipoo signaling pathway)	(99)
		BTV	Testis primary cells	↑ let-7d, miR-29b, mir-29, mir-61<br><br>↓let-7f, mir-10b, miR-369-5p, mir-158, mir-805	Genes involve in MAPK, PI3K-Akt, endocytosis, Hippo, NF-kB, viral carcinogenesis, FoxO, JAK-STAT and TLR signaling pathways	(100)
		TSE prion		↑miR-342-3p, miR-21-5p		(102)
		Echinococcusgran ulosus	Intestinal tissue	↑miR-21-3p, miR-542-5p, miR-671, miR-134-5p, miR-26b, miR-27a	Genes involved in the inflammation process (NF-kB pathway-responsive)	(103)

	Capra hircus	PPRV	PMBC	miR-664, miR-2311, miR-2897, miR-484, miR-2440, miR-3533, miR-574<br><br>↓miR-218, miR-210, miR-21-5p, miR-30	Genes involved in proviral and antiviral activities: immune response, apoptosis and cells sensitivity to the antiviral activity of interferon.<br>MiR-218 directly targeted *SLAM (or CD150*); miR-21-5p directly targeted *TGFBR2*	(95) (96)
			Spleen	↑miR-17-3p, miR-486, miR-146b, miR-363, miR-451, miR-193a-3p, miR-760-3p, miR-144, miR-21-5p<br><br>↓miR-199b, miR-1271, miR-217, miR-2887-1, miR-2887-2, miR-6119-3p, miR-221, miR-744, miR-30c, let-7a-5p-2, miR-211	Genes involved in immune response and apoptosis signalling pathways	(97)
			Lung	↑miR-320a-1, miR-320a-2, miR-1246, miR-363, miR-760-3p, miR-21-3p<br><br>↓miR-34b, miR-150		(97)
Colostrum and milk	*Ovis aries*	pregnancy	Mammary gland	↑ miR-21, miR-200, miR-205	tumour-suppressor phosphatase and tensin homolog (*PTEN*); E-cadherin	(100)

					transcriptional repressors *ZEB1* (also known as δEF1) and *SIP1* (also known as ZEB2)	
				↓miR-432, miR-200b, miR-29	These miRNAs are involved in miRNA-circRNA-mRNA network. Putative target genes are *ZEB1*and *ZEB2*, genes involved in Wnt/β-catenin signaling and in secretion of lactoprotein, triglycerides, and lactose.	(108)
	Capra hircus	N/A	Lactating mammary gland (early- peak - late lactation)	↑miR-423-5p, miR-378, miR-7	Genes involved in lactation regarding milk ingredient transport and ingredient synthesis	(105)
			Lactating mammary gland (colostrum/milk)	↑miR-574<br><br>Common expression on both matrices: let-7a, let-7b, let-7c, let-7f, let-7g, miR-21, miR-230a, miR-2103, miR-2107, miR-2143, miR-2148a, miR-2320, miR-2423-5p	Genes involved in oestrogen, endocrine, adipocytokine (*LEPR*), oxytocin (*PPP1CA*) and MAPK signaling pathways	(106)
			Lactating mammary gland	miR-2887, miR-29a, miR-30a, miR-101, miR-146b	Genes involved in lactation regulation, IFN-γ, NF-κB and ICOS signaling	(109)

Management stress	*Capra hircus*	Weaning	Serum	↓miR-206 and miR-133a/b miR-99b-3p, miR-224, miR-143-5p, miR-10b-5p	Genes involved in skeletal muscle development and in cell proliferation-associated pathway	(111)
		Diet changes	*Hypothalamus* and ovary	↑ in hypothalamus ↓in ovary miR-200a, miR-200b, miR-200c	Genes involved in regulation of estrus-related genes (*ITPR*, *PRKCB*, *GPR54 and KISS1*) and in the HPO axis through *GNAQ* gene in the hypothalamus	(113)
		Food deprivation	Mammary gland	↑miR-99a-5p, miR-126-3p, miR-140-3p, miR-222-3p, miR-223-3p, miR-204-5p, miR-409-3p	Genes involved in lipid metabolism pathway	(114)
Environmental stress	*Capra hircus*	High altitude hypoxia	heart, kidney, liver, muscle, spleen	miR-106-5p<br>↓ miR-509-3p, miR-3069-1-3p, miR-409-5p, miR-208-3p	Genes involved in apoptosis, angiogenesis, DNA damage repair, erythropoiesis, and energy metabolism. *VEGFR1* (or *FLT1*) and HIF1 pathway	(115)

N/A= not applicable

PPRV: Peste des petits ruminants virus

VMV: Visna Maedy Virus

SPPV: Sheep pox virus

BTV: Bluetongue Virus

TSE: Transmissible spongiform encephalopathie

PMBC: peripheral blood mononuclear cells
