# Supplementary material for: MicroRNAs as Biomarkers for Animal Health and Welfare in Livestock
Source: Front Vet Sci. 2020 Dec 18;7:578193. doi: 10.3389/fvets.2020.578193 (PMC7775535; doi:10.3389/fvets.2020.578193)
Supplement: Supplementary file 3 [file Table_3.pdf]

Supplementary Material

Table 3_Putative biomarkers and DE-miRNAs for experienced stress or stress susceptibility in swine specie

	Model/disease	Target organ-tissues	DE-miRNAs Modulation	Predicted target	Reference
Endocrine system	HPA axis activation by corticotropin-releasing-factor (CRF)	Adrenal gland	↓miR-375	*specificity protein 1 (SP1)*	(117)
	Gonadotropin-releasing hormone (GnRH)	Pituitary gland	↓miR-361-3p	*Follicle Stimulating Hormone β subunit (FSHB)*	(118)
	Zearalenone (ZEA) mycotoxin	Pituitary gland	↑ miR-7	FSH synthesis and secretion inhibition by targeting *FOS*	(119)
	Growth hormone releasing hormone (GHRH) and cortistatin (CST)	Pituitary gland	↑let-7c	Growth hormone (*GH1*) and GHRH receptor (*GHRHR*)	(120)
Immunity	influenza A virus (H1N2)	Lung Leucocytes	↑miR-15a, miR-18a, miR-21, miR-29b, miR-590-3p	*BCL2*, *MCL1*, *EIF2AK2* and genes involved in pro and anti-inflammatory cytokines, apoptosis	(121, 122)
	Reproductive Respiratory Syndrome Virus (PRRSV)	Alveolar macrophage	↓miR-29, miR-27b-3p, miR-26<br><br>↑ miR-23, miR-378, miR-505	*Nsp2*, and genes involved in IFN pathway (MX1, IFI44), chemokine and cytokine<br><br>*AKT3*, *TP53INP1*, *RPS6KB1*, *JAK1*	(123, 125, 128, 129, 130)
	African Swine Fevere Virus (ASFV)	Spleen Lymph nodes	↓ miR-125b, miR-451, miR-125a	Genes involved in immune response (B and T cell receptor signaling pathway, natural killer	(132)

			↑miR-126-5p, miR-92c, miR-92a, miR-30e-5p, miR-500a-5p	cell mediated cytotoxicity or Fc gamma R-mediated phagocytosis)	
	Foot and Mouth Disease Virus (FMDV)	Porcine kidney cell line (PK-15)	↑miR-1307	Genes involved in enhancing host immune response and suppressing viral replication (virus structural protein VP3)	(126)
	Porcine Epidemic diarrhea virus (PEDV)	African green monkey kidney cells (MARC-145)	↑miR-221-5p	Genes involved in enhancing host immune response and suppressing viral replication (NF-κB-inhibitor α and suppressor of cytokine signaling 1; virus replication protein PEDV)	(124)
Colostrum and Milk	Healthy suckling piglets	Colostrum *vs* milk exosomes Blood	↑miR-148a-3p, miR-182-5p, miR-200c-3p, miR-25-3p, miR-30a-5p, miR-30d-5p, miR-574-3p	Genes withroles in the development of the immune system	(135)
	Healthy lactating pigs	Milk exosomes	miR-191, let-7a, miR-193a-3p, miR-423-5p, miR-320, miR-181a, miR-30a-3p, miR-378, let-7f, let-7c<br>N/A modulation	Genes involved in immunity and metabolism processes, including the intestinal immune network for IgA production, antigen processing and presentation.	(136)
	Ginseng polysaccharides supplementation	Milk exosomes	↓ let-7d<br>↑ miR-30a	Genes involved in regulating immune response	(137)
	Lipopolysaccharide (LPS) endotoxin	Intestinal porcine enterocytes (IPEC-J2 cell line)	↑miR-219, miR-338, miR-4334	*TRL4*, *MyD88*, *Tp53*(genes involved in p53 pathway)	(138)

Management stress	Tail docking	Saliva	↑miR-19b, miR-27b-3p, miR-215, miR-22-3p, miR-155-5p, miR-365-5p, miR-204	Genes involved in inflammatory responce	(24)
	Weaning	Jejunum Serum	↓miR-30c-5p, miR-144, miR-150, miR-186, miR-194a, miR-194b-5p, miR-363<br><br>↑miR-21, miR-31, miR-205	*Small ubiquitin-related modifier-2 (SUMO-2)*pivotal for cell homeostasis during endogenous or environmental stress (heat shock and nutrient stress)	(139)
Environmental stress	Heat	Skeletal muscle	↓ miR-486, miR-133a, miR-191, miR-181a, miR-27b-3p<br><br>↑ miR-183, miR-30e-5p, miR-1468	Genes involved in functional categories of skeletal muscle structure and function, nutrient utilization and stress response pathway	(155)

N/A= not applicable
