# Supplementary material for: MicroRNAs as Biomarkers for Animal Health and Welfare in Livestock
Source: Front Vet Sci. 2020 Dec 18;7:578193. doi: 10.3389/fvets.2020.578193 (PMC7775535; doi:10.3389/fvets.2020.578193)
Supplement: Supplementary file 4 [file Table_4.pdf]

Supplementary Material

Table 4_DE-miRNAs immunity-related for experienced stress or stress susceptibility in swine specie

miRNA	Modulation	Patho-pysiological condition	Matrix	Approch	Functions	Reference
**let-7f-5**	N/A	Healthy	Exosomes: urine, plasma, semen, bile	Illumina HiSeqTM 2502	immunity and metabolis	(1)
**let-7i-5**	N/A	Healthy	Exosomes: urine, plasma, semen, bile	Illumina HiSeqTM 2503	immunity and metabolis	(1)
**let-7g**	↑	PRRSV	PAMs	IlluminaHiseq 2000	inibition virus replication	(2)
**let-7c**	↓	*Toxoplasma gondii*	spleen	IlluminaHiseq 2519	immune response, metabolism, diseases	(3)
**let-7a**	↓	*Toxoplasma gondii*	spleen	IlluminaHiseq 2522	immune response, metabolism, diseases	(3)
**let-7d-3p**	↑	*Trichuris suis*	circulating - serum	RT-qPCR	Immune response by targeting IL13	(4)
**miR-122**	↑	*Toxoplasma gondii*	spleen	Illumina Hiseq 2507	immune response, metabolism, diseases	(3)

	↑	Healthy Yorkshire *vs* Queshan Black	spleen	Illumina HiSeqTM 2002	immune response e disease resistance	(5)
	↑	African swine fever virus	Spleen, submandibular lymph node	Ion Torrent PGM sequencer	immune response, virus-host interactions	(6)
**miR-10b**	↑	PRRSV	PAMs	Illumina Hiseq 2000	inhibition virus replication	(2)
**miR-125b**	↑	Lawsonia intracellularis	ileum	GeneChip® miRNA 4.0 Array Affymetrix	immunity, metabolism and cellular processes	(7)
	↑	PRRSV	MARC-145 cells	RT-qPCR	inhibition of virus infection targeting NF-kB	(8)
**miR-126-5p**	↓	African swine fever virus	Spleen, submandibular lymph node	Ion Torrent PGM sequencer	immune response, virus-host interactions	(6)
**miR-126-3p**	↓	African swine fever virus	Spleen, submandibular lymph node	Ion Torrent PGM sequencer	immune response, virus-host interactions	(6)
**miR-127**	↑	*Toxoplasma gondii*	spleen	Illumina Hiseq 2504	immune response, metabolism, diseases	(3)
	↓	PRRSV	MARC-145 cells	RT-qPCR	immune response	(9)
**miR-129a-3p**	↓	*Toxoplasma gondii*	spleen	Illumina Hiseq 2516	immune response, metabolism, diseases	(3)

**miR-1307**	N/A	foot-and-mouth disease virus replication	porcine kidney cell line PK-15	Affymetrix miRNA array analysis (miRNA-4_0)	suppression virus replication, enhance host immune response	(10)
**miR-133a**	↑	Aujeszky's disease virus suis herpesvirus type 1 [SuHV-1]	Olfactory bulb and trigeminal ganglia	315 chips in Ion PGM™ sequencer	host-pathogen interaction	(11)
**miR-133b**	↑	Aujeszky's disease virus suis herpesvirus type 1 [SuHV-1]	Olfactory bulb (OB) and trigeminal ganglia (TG)	316 chips in Ion PGM™ sequencer	host-pathogen interaction	(11)
**miR-143-3p**	↑	Lawsonia intracellularis	ileum	GeneChip® miRNA 4.0 Array Affymetrix	immunity, metabolism and cellular processes	(7)
**miR-145**	↑	African swine fever virus	Spleen, submandibular lymph node	IonTorrent PGM sequencer	immune response, virus-host interactions	(6)
	↑	PRRSV	MARC-145 cells	RT-qPCR	immune response	(9)
**miR-146**	↑	PRRSV	PAMs	Illumina Hiseq 2000	inhibition virus replication	(2)
	↑	influenza A (H1N2)	leukocytes	high-throughput RT-qPCR platform BioMark (Fluidigm)	apoptosis and cell cycle regulation; predispose to secondary infection	(12)
	↓	influenza A (H1N2)	lungs	RT-qPCR	controlling influenza infection	(13)

**miR-148a-3p**	N/A	healthy	exosomes: urine, plasma, semen, bile	IlluminaHiSeqTM 2500	immunity and metabolis	(1)
**miR-149**	↓	*Toxoplasma gondii*	spleen	Illumina Hiseq 2512	immune response, metabolism, diseases	(3)
**miR-150-5p**	↓	influenza A (H1N2)	leukocytes	high-throughput RT-qPCR platform BioMark (Fluidigm)	apoptosis and cell cycle regulation; predispose to secondary infection	(12)
**miR-155**	↓	Healthy Yorkshire vsQueshan Black pigs	spleen	IlluminaHiSeqTM 2008	immune response and disease resistance	(5)
	↑	Inflammation	PK-15 cells stimulated with LPS	RT-qPCR	Activation of the TLR3/TLR4 signalling pathways	(14)
**miR-15a**	↑	influenza A (H1N2)	lung	IlluminaNextSeq 500 system	modulators of viral pathogen recognition and apoptosis	(15)
	↑	influenza A (H1N2)	leukocytes	high-throughput RT-qPCR platform BioMark (Fluidigm)	apoptosis and cell cycle regulation; predispose to secondary infection	(12)
	↑	influenza A (H1N2)	lung	RT-qPCR	controlling influenza infection	(13)
**miR-16-5p**	↓	influenza A (H1N2)	leukocytes	high-throughput RT-qPCR platform BioMark (Fluidigm)	apoptosis and cell cycle regulation; predispose to secondary infection	(12)

**miR-17-5p**	↑	*Toxoplasma gondii*	spleen	Illumina Hiseq 2509	immune response, metabolism, diseases	(3)
**miR-18a**	↑	Influenza A (H1N2)	lung	Illumina NextSeq 500 system	modulators of viral pathogen recognition and apoptosis	(15)
**miR-181a**	↑	African swine fever virus	Spleen, submandibular lymph node	Ion Torrent PGM sequencer	immune response, virus-host interactions	(6)
	↑	Lawsoniaintracellularis	ileum	GeneChip® miRNA 4.0 Array Affymetrix	immunity, metabolism and cellular processes	(7)
**miR-182**	↑	*Toxoplasma gondii*	spleen	Illumina Hiseq 2508	immune response, metabolism, diseases	(3)
**miR183**	↑	Inflammation	monocytes stimulated with LPS	Microarrays miRNA Probe Set V15, Capitalbio	regulation of NF-kB and MyD88 = suppression of TLRs	(16)
**miR-184**	↓	Healthy Yorkshire vsQueshan Black pigs	spleen	IlluminaHiSeqTM 2014	immune response e disease resistance	(5)
**miR-185**	↑	*Toxoplasma gondii*	spleen	IlluminaHiseq 2501	immune response, metabolism, diseases	(3)
**miR-186**	↑	Influenza A (H1N2)	leukocytes	high-throughput RT-qPCR platform BioMark (Fluidigm)	apoptosis and cell cycle regulation; predispose to secondary infection	(12)

**miR-194b**	↓	Lawsonia intracellularis	ileum	GeneChip® miRNA 4.0 Array Affymetrix	immunity, metabolism and cellular processes	(7)
**miR-199b-5p**	↑	Healthy Yorkshire vs Queshan Black pigs	spleen	IlluminaHiSeqTM 2005	immune response e diseaseresistance	(5)
**miR-203a-3p**	↑	influenza A (H1N2)	leukocytes	high-throughput RT-qPCR platform BioMark (Fluidigm)	apoptosis and cell cycle regulation; predispose to secondary infection	(12)
**miR-206**	↑	Aujeszky's disease virus suis herpesvirus type 1 [SuHV-1]	Olfactory bulb and trigeminal ganglia	314 chips in Ion PGM™ sequencer	host-pathogen interaction	(11)
	↑	influenza A (H1N2)	lungs	RT-qPCR	controlling influenza infection	(13)
**miR-20a**	↑	*Toxoplasma gondii*	spleen	Illumina Hiseq 2510	immune response, metabolism, diseases	(3)
**miR-21**	↓	influenza A (H1N2)	lung	IlluminaNextSeq 500 system,	modulators of viral pathogen recognition and apoptosis.	(15)
	↑	influenza A (H1N2)	lung	RT-qPCR	controlling influenza infection	(13)
	N/A	healthy	exosomes: urine, plasma, semen, bile	IlluminaHiSeqTM 2501	immunity and metabolis	(1)

**miR-210**	↑	*Toxoplasma gondii*	spleen	Illumina Hiseq 2505	immune response, metabolism, diseases	(3)
**miR-214**	↑	Lawsonia intracellularis	ileum	GeneChip® miRNA 4.0 Array Affymetrix	immunity, metabolism and cellular processes	(7)
**miR-215**	↓	Lawsonia intracellularis	ileum	GeneChip® miRNA 4.0 Array Affymetrix and qPCR	immunity, metabolism and cellular processes	(7)
**miR-219a**	↑	Healthy Yorkshire vs Queshan Black pigs	spleen	Illumina HiSeqTM 2000	immune response e diseas eresistance	(5)
**miR-221-5p**	↑	Porcine epidemic diarrhea virus (PEDV)	African green monkey kidney cells, MARC-145 and IECs	RT-qPCR	inhibition virus replication, activation of NF-kB and IFN beta	(17)
**miR-223**	↓	influenza A (H1N2)	leukocytes	high-throughput RT-qPCR platform BioMark (Fluidigm)	apoptosis and cell cycle regulation; predispose to secondary infection	(12)
	↑	influenza A (H1N2)	lung	RT-qPCR	controlling influenza infection	(13)
**miR-2320-5p**	↓	*Toxoplasma gondii*	spleen	IlluminaHiseq 2517	immune response, metabolism, diseases	(3)
	↑	Healthy Yorkshire vs Queshan Black pigs	spleen	IlluminaHiSeqTM 2006	immune response, disease resistance	(5)

**miR-22-5p**	↑	influenza A (H1N2)	leukocytes	high-throughput RT-qPCR platform BioMark (Fluidigm)	apoptosis and cell cycle regulation; predispose to secondary infection	(12)
**miR-23b**	↓	African swine fever virus	Spleen, submandibular lymph node	Ion Torrent PGM sequencer	immune response, virus-host interactions	(6)
	↓	influenza A (H1N2)	leukocytes	high-throughput RT-qPCR platform BioMark (Fluidigm)	apoptosis and cell cycle regulation; predispose to secondary infection	(12)
**miR-23a**	↓	influenza A (H1N2)	leukocytes	high-throughput RT-qPCR platform BioMark (Fluidigm)	apoptosis and cell cycle regulation; predispose to secondary infection	(12)
	↑	PRRSV	MARC-145 cells	RT-qPCR	inhibition of virus infection affecting IFN type I	(18)
	↓	African swine fever virus	Spleen, submandibular lymph node	Ion Torrent PGM sequencer	immune response, virus-host interactions	(6)
**miR-26a**	↑	PRRSV	MARC-145 cells	RT-qPCR	promotion innate anti-viral responses	(19)
	↑	PRRSV	MARC-145 cells	RT-qPCR	inhibition of virus infection affecting IFN type I	(20)
**miR-27b**	↓	PRRSV	PAMs	Illumina Hiseq 2000	inhibition virus replication	(2)

	↑	Inflammation	monocytes stimulated with LPS	Microarrays miRNA Probe Set V15, Capitalbio	regulation of NF-kB and MyD88 = suppression of TLRs	(16)
**miR-27a***	↓	Healthy Yorkshire vs Queshan Black pigs	spleen	Illumina HiSeqTM 2015	immune response e disease resistance	(5)
**miR-28-5p**	↑	influenza A (H1N2)	leukocytes	high-throughput RT-qPCR platform BioMark (Fluidigm)	apoptosis and cell cycle regulation; predispose to secondary infection	(12)
**miR-296-3p**	↓	*Toxoplasma gondii*	spleen	Illumina Hiseq 2514	immune response, metabolism, diseases	(3)
**miR-296-5p**	↓	Healthy Yorkshire vs Queshan Black pigs	spleen	Illumina HiSeqTM 2011	immune response e disease resistance	(5)
**miR-29b**	↑	influenza A (H1N2)	lung	Illumina NextSeq 500 system	modulators of viral pathogen recognition and apoptosis	(15)
	↑	influenza A (H1N2)	leukocytes	high-throughput RT-qPCR platform BioMark (Fluidigm)	apoptosis and cell cycle regulation; predispose to secondary infection	(12)
**miR-29a**	↑	influenza A (H1N2)	leukocytes	high-throughput RT-qPCR platform BioMark (Fluidigm)	apoptosis and cell cycle regulation; predispose to secondary infection	(12)
	↑	PRRSV	PAMs	RT-qPCR	promotion of virus replication	(21)

**miR-30b-3p**	↓	*Toxoplasma gondii*	spleen	Illumina Hiseq 2513	immune response, metabolism, diseases	(3)
**miR-30d**	↓	African swine fever virus	Spleen, submandibular lymph node	Ion Torrent PGM sequencer	immune response, virus-host interactions	(6)
**miR-328**	↓	Toxoplasma gondii	spleen	Illumina Hiseq 2518	immune response, metabolism, diseases	(3)
**miR-331-3p**	↓	Healthy Yorkshire vs Queshan Black pigs	spleen	Illumina HiSeqTM 2017	immune response e disease resistance	(5)
**miR-335**	↑	inflammation	monocytes stimulated with LPS	Microarrays miRNA Probe Set V15, Capitalbio	regulation of NF-kB and MyD88 = suppression of TLRs	(16)
**miR-339-5p**	↓	Healthy Yorkshire vs Queshan Black pigs	spleen	Illumina HiSeqTM 2009	immune response e disease resistance	(5)
	↓	Healthy Yorkshire vs Queshan Black pigs	spleen	Illumina HiSeqTM 2012	immune response e disease resistance	(5)
**miR-342**	↓	Lawsonia intracellularis	ileum	GeneChip® miRNA 4.0 Array Affymetrix and qPCR	immunity, metabolism and cellular processes	(7)
**miR-361-3p**	↑	*Toxoplasma gondii*	spleen	Illumina Hiseq 2500	immune response, metabolism, diseases	(3)

**miR-378b-3p**	↓	Healthy Yorkshire vs Queshan Black pigs	spleen	Illumina HiSeqTM 2013	immune response e disease resistance	(5)
	↓	inflammation	monocytes stimulated with LPS	Microarrays miRNA Probe Set V15, Capitalbio	regulation of NF-kB and MyD88 = suppression of TLRs	(16)
**miR-378**	↑	PRRSV	MARC-145 cells	RT-qPCR	inhibition of virus infection affecting IFN type I	(18)
	↑	Aujeszky's disease virus suis herpesvirus type 1 [SuHV-1]	Olfactory bulb and trigeminal ganglia	317 chips in Ion PGM™ sequencer	host-pathogen interaction	(11)
**miR-4332***	↑	Healthy Yorkshire vs Queshan Black pigs	spleen	Illumina HiSeqTM 2001	immune response e disease resistance	(5)
**miR-4334-3p***	↓	Healthy Yorkshire vs Queshan Black pigs	spleen	Illumina HiSeqTM 2018	immune response e disease resistance	(5)
**miR-449a**	↑	influenza A (H1N2)	leukocytes	high-throughput RT-qPCR platform BioMark (Fluidigm)	apoptosis and cell cycle regulation; predispose to secondary infection	(12)
**miR-451**	↑	influenza A (H1N2)	lung	RT-qPCR	controlling influenza infection	(13)
	↑	African swine fever virus	Spleen, submandibular lymph node	Ion Torrent PGM sequencer	immune response, virus-host interactions	(6)

**miR-455-5p**	↑	Healthy Yorkshire vs Queshan Black pigs	spleen	IlluminaHiSeqTM 2004	immune response e disease resistance	(5)
**miR-486**	↓	inflammation	monocytes stimulated with LPS	Microarrays miRNA Probe Set V15, Capitalbio	regulation of NF-kB and MyD88 = suppression of TLRs	(16)
	↑	*Toxoplasma gondii*	spleen	Illumina Hiseq 2503	immune response, metabolism, diseases	(3)
**miR-493-5p**	↓	*Toxoplasma gondii*	spleen	Illumina Hiseq 2521	immune response, metabolism, diseases	(3)
**miR-500**	↑	*Toxoplasma gondii*	spleen	Illumina Hiseq 2502	immune response, metabolism, diseases	(3)
**miR-504**	↓	*Toxoplasma gondii*	spleen	Illumina Hiseq 2515	immune response, metabolism, diseases	(3)
	↓	Healthy Yorkshire vs Queshan Black pigs	spleen	Illumina HiSeqTM 2016	immune response e disease resistance	(5)
**miR-505**	↑	PRRSV	MARC-145 cells	RT-qPCR	inhibition of virus infection affecting IFN type I	(18)
**miR-542-3p**	↑	*Toxoplasma gondii*	spleen	Illumina Hiseq 2511	immune response, metabolism, diseases	(3)
**miR-628**	↑	inflammation	monocytes stimulated with LPS	Microarrays miRNA Probe Set V15, Capitalbio	regulation of NF-kB and MyD88 = suppression of TLRs	(16)

**miR-7138-5p**	↓	*Toxoplasma gondii*	spleen	Illumina Hiseq 2520	immune response, metabolism, diseases	(3)
**miR-769-3p**	↑	Healthy Yorkshire vs Queshan Black pigs	spleen	Illumina HiSeqTM 2003	immune response and disease resistance	(5)
**miR-92a**	↓	African swine fever virus	Spleen, submandibular lymph node	Ion Torrent PGM sequencer	immune response, virus-host interactions	(6)
**miR-92b-3p**	↓	African swine fever virus	Spleen, submandibular lymph node	Ion Torrent PGM sequencer	immune response, virus-host interactions	(6)
**miR-92c**	↓	African swine fever virus	Spleen, submandibular lymph node	Ion Torrent PGM sequencer	immune response, virus-host interactions	(6)
**miR-9820-5p**	↓	Healthy Yorkshire vs Queshan Black pigs	spleen	Illumina HiSeqTM 2010	immune response and disease resistance	(5)
**miR-99a-5p**	N/A	healthy	exosomes: urine, plasma, semen, bile	Illumina HiSeqTM 2504	immunity and metabolis	(1)
	↓	PRRSV	PAMs	Illumina Hiseq 2000	inibition virus replication	(2)
**novel-154**	↑	*Toxoplasma gondii*	spleen	Illumina Hiseq 2506	immune response, metabolism, diseases	(3)
**novel-262**	↓	*Toxoplasma gondii*	spleen	Illumina Hiseq 2523	immune response, metabolism, diseases	(3)

PAMs= porcine alveolar macrophages

PRRSV= Porcine Reproductive and Respiratorysyndrome virus

N/A= not applicable

Bibliography

1. Zhang J, Luo H, Xiong Z, Wan K, Liao Q, He H. High-throughput sequencing reveals biofluid exosomal miRNAs associated with immunity in pigs. *Biosci Biotechnol Biochem* (2020) **84**:53–62. doi:10.1080/09168451.2019.1661767

2. Wu J, Ji Z, Qiao M, Peng X, Wu H, Song Z, Zhao H, Liu G, Li F, Mei S. MicroRNA transcriptome analysis of poly I:C-stimulated and PRRSV-infected porcine alveolar macrophages. *J Appl Genet* (2019) **60**:375–383. doi:10.1007/s13353-019-00500-3

3. Hou Z, Liu D, Su S, Wang L, Zhao Z, Ma Y, Li Q, Jia C, Xu J, Zhou Y, et al. Comparison of splenocyte microRNA expression profiles of pigs during acute and chronic toxoplasmosis. *BMC Genomics* (2019) **20**:97. doi:10.1186/s12864-019-5458-y

4. Hansen EP, Kringel H, Thamsborg SM, Jex A, Nejsum P. Profiling circulating miRNAs in serum from pigs infected with the porcine whipworm, Trichuris suis. *Vet Parasitol* (2016) **223**:30–3. doi:10.1016/j.vetpar.2016.03.025

5. Li X, Qiao R, Ye J, Wang M, Zhang C, Lv G, Wang K, Li X, Han X. Integrated miRNA and mRNA transcriptomes of spleen profiles between Yorkshire and Queshan black pigs. *Gene* (2019) **688**:204–214. doi:10.1016/j.gene.2018.11.077

6. Núñez-Hernández F, Pérez LJ, Muñoz M, Vera G, Accensi F, Sánchez A, Rodríguez F, Núñez JI. Differential expression of porcine microRNAs in African swine fever virus infected pigs: a proof-of-concept study. *Virol J* (2017) **14**:198. doi:10.1186/s12985-017-0864-8

7. Li H, Zhang M, Zheng E. Comprehensive miRNA expression profiles in the ilea of Lawsonia intracellularis-infected pigs. *J Vet Med Sci* (2017) **79**:282–289. doi:10.1292/jvms.16-0423

8. Wang D, Cao L, Xu Z, Fang L, Zhong Y, Chen Q, Luo R, Chen H, Li K, Xiao S. MiR-125b reduces porcine reproductive and respiratory syndrome virus replication by negatively regulating the NF-κB pathway. *PLoS One* (2013) **8**:e55838. doi:10.1371/journal.pone.0055838

9. Zhou A, Li S, Zhang S. miRNAs and genes expression in MARC-145 cell in response to PRRSV infection. *Infect Genet Evol* (2014) **27**:173–80. doi:10.1016/j.meegid.2014.07.023

10. Qi L, Wang K, Chen H, Liu X, Lv J, Hou S, Zhang Y, Sun Y. Host microRNA miR-1307 suppresses foot-and-mouth disease virus replication by promoting VP3 degradation and enhancing innate immune response. *Virology* (2019) **535**:162–170. doi:10.1016/j.virol.2019.07.009

11. Timoneda O, Núñez-Hernández F, Balcells I, Muñoz M, Castelló A, Vera G, Pérez LJ, Egea R, Mir G, Córdoba S, et al. The role of viral and host microRNAs in the Aujeszky's disease virus during the infection process. *PLoS One* (2014) **9**: doi:10.1371/journal.pone.0086965

12. Brogaard L, Heegaard PMH, Larsen LE, Mortensen S, Schlegel M, Dürrwald R, Skovgaard K. Late regulation of immune genes and microRNAs in circulating leukocytes in a pig model of influenza A (H1N2) infection. *Sci Rep* (2016) **6**: doi:10.1038/srep21812

13. Skovgaard K, Cirera S, Vasby D, Podolska A, Breum SO, Dürrwald R, Schlegel M, Heegaard PM. Expression of innate immune genes, proteins and microRNAs in lung tissue of pigs infected experimentally with influenza virus (H1N2). *Innate Immun* (2013) **19**:531–544. doi:10.1177/1753425912473668

14. Li C, He H, Zhu M, Zhao S, Li X. Molecular characterisation of porcine miR-155 and its regulatory roles in the TLR3/TLR4 pathways. *Dev Comp Immunol* (2013) **39**:110–6. doi:10.1016/j.dci.2012.01.001

15. Brogaard L, Larsen LE, Heegaard PMH, Anthon C, Gorodkin J, Dürrwald R, Skovgaard K. IFN-λ and microRNAs are important modulators of the pulmonary innate immune response against influenza A (H1N2) infection in pigs. *PLoS One* (2018) **13**:e0194765. doi:10.1371/journal.pone.0194765

16. Jun H, Ying H, Daiwen C, Bing Y, Xiangbing M, Ping Z, Jie Y, Zhiqing H, Junqiu L. MIR-628, a microRNA that is induced by Toll-like receptor stimulation, regulates porcine innate immune responses. *Sci Rep* (2015) **5**: doi:10.1038/srep12226

17. Zheng H, Xu L, Liu Y, Li C, Zhang L, Wang T, Zhao D, Xu X, Zhang Y. MicroRNA-221-5p Inhibits Porcine Epidemic Diarrhea Virus Replication by Targeting Genomic Viral RNA and Activating the NF-κB Pathway. *Int J Mol Sci* (2018) **19**: doi:10.3390/ijms19113381

18. Zhang Q, Guo X kun, Gao L, Huang C, Li N, Jia X, Liu W, Feng W hai. MicroRNA-23 inhibits PRRSV replication by directly targeting PRRSV RNA and possibly by upregulating type I interferons. *Virology* (2014) **450–451**:182–195. doi:10.1016/j.virol.2013.12.020

19. Jia X, Bi Y, Li J, Xie Q, Yang H, Liu W. Cellular microRNA miR-26a suppresses replication of porcine reproductive and respiratory syndrome virus by activating innate antiviral immunity. *Sci Rep* (2015) **5**: doi:10.1038/srep10651

20. Li L, Wei Z, Zhou Y, Gao F, Jiang Y, Yu L, Zheng H, Tong W, Yang S, Zheng H, et al. Host miR-26a suppresses replication of porcine reproductive and respiratory syndrome virus by upregulating type I interferons. *Virus Res* (2015) **195**:86–94. doi:10.1016/j.virusres.2014.08.012

21. Zhou M, Li C, Lu C, Zhang X, Pan Y, Liu X, Liu G, Zhao Z, Sun B. MiRNA29 Promotes Viral Replication during Early Stage of PRRSV Infection in Vitro. *DNA Cell Biol* (2016) **35**:636–642. doi:10.1089/dna.2015.3103
