# Supplementary material for: MicroRNAs as Biomarkers for Animal Health and Welfare in Livestock
Source: Front Vet Sci. 2020 Dec 18;7:578193. doi: 10.3389/fvets.2020.578193 (PMC7775535; doi:10.3389/fvets.2020.578193)
Supplement: Supplementary file 5 [file Table_5.pdf]

Supplementary Material

Table 5_Putative biomarkers and DE-miRNAs for experienced stress or stress susceptibility in poultry species

	Model/disease	Target organ-tissues	DE-miRNAs Modulation	Predicted target	Reference
Endocrine system	Tonic immobility	Hypothalamus	↓ miR-181, miR-211, miR-22	Glucocorticoid receptor	(157)
Immunity	IBV	Spleen	↓ miR-34c-5p, miR-34b-5p, miR-1788-3p, miR-203a, miR-200a-3p, miR-200b-3p, miR-429-3p, miR-135a-5p, miR-205a-5p, miR-187-3p, miR-1a-3p, miR-122-5p, miR-449a-5p, miR-449c-5p, miR-133c-3p, miR-133a-3p	NFATC3, NFAT5, SPPL3, NOTCH1, JUN, TAB2, TGFB2	(158)
		Lungs	↓ miR-206, miR-499-5p, miR-133b, miR-1a-3p, miR-122-5p	NFATC3, NFAT5, SPPL3, SPREAD, TAOK3, VAV3	
		Kidney	↓ miR-30d<br>↑ miR-1a-3p<br>regulation associated with IBV pathogenicity: miR-215-5p, miR-1454, miR-7b, miR-3538 and miR-2954	Genes involved in immune responses, the regulation of cellular process	(159)
		Bone marrow-derived dendritic cells	↑ miR-135a, miR-7471, miR-7453, miR-7443, miR-1695, miR-1772, and miR-6669<br>↓ miR-6632-5p, miR-7467-3p, miR-449b-3p, miR-6658-3p, miR-2131-3p, miR-34c-3p, miR-1694, miR-3535,	Genes involved in actin cytoskeleton and MAPK signal pathway	(160)

			miR-21-5p, miR-1462-3p, miR-6656-5p, miR-6651-5p		
	ALV-J	Spleen	miR-205a, miR-21-5p, miR-21-3p, miR-383-5p, miR-203, miR-223 and miR-148a-5p	Genes connected to immune response	(162)
		Bone marrow-derived dendritic cells	↓ miR-221, miR-211, miR-222a<br><br>↑ miR-125b,miR-193b, miR-148a, miR-27b, miR-34a, miR-130a	Genes involved in antigen presentation, apoptosis	(163)
		Spleen	↑ miR-34b-5p, miR-23b	Genes involved in the interferon pathway, melanoma differentiation-associated gene 5 (*MDA5*) and interferon regulatory factor 1	(163) (164)
	REV	Spleen	↓ miR-200a-3p<br><br>↑ miR-375<br><br>miR-1458, miR-1664-3p, miR-122-5p, miR-222b-5p, miR-147, miR-1329-5p, miR-1618-5p, miR-1664-3p, miR-146b-3p, miR-222b-3p, miR-144-3p, and miR-1769-3 (not specified)	Genes involved in endocrine system, immune system, cell growth and death	(165)
	DHAV-3	Liver	↓ miR-32-5p, miR-125-5p, miR-128-3p, miR-460-5p, novel-m0012-3p	Genes involved in cytokine-cytokine receptor interaction, apoptosis, Toll-like receptor, FoxO and Jak-STAT signalling pathways	(166)
Management and stress	Feed deprivation	Liver	↓ miR-33<br><br>↑ miR-20b, miR-34a, and miR-454	*FADS1*, *FOXO3*	(170)
	Selenium deprivation	Cardiomyocytes	↑ miR-200a-5p, miR-2954	Genes involved in necroptosis, autophagy and apoptosis (PI3K pathway)	(171, 172)
		Skeletal muscle	↑ let-7f-3p	*selenoprotein K*	(173)
		Chondrocytes	↑ miR-138-5p	*caspase 3* and *9*, *BAX* and *BAK*, and *selenoprotein M*	(174)

	Manganese supplementation during thermal stress	Hearts of offspring embryos	↑miR-1551 and miR-34b, miR-34c, miR-31	*BCL2* and *NF-kB*	(176)
Environmental stress	Heat stress	Plasma	↑ miR-2130, miR-92-5p, miR-1618-5p, miR-3064-3p, miR-6575-5p, miR-1737, miR-3525, miR-10a-3p, miR-6557-5p, miR-6568-3p, miR-6548-5p<br><br>↓ miR-15b-3p, miR-1808, miR-202-5p, miR-9-3p, miR-6642-5p	Genes involved in the cellular response to stress and lipid metabolism	(180)
		Duck duodenum, jejunum and ileum	16 DE-miRNAs<br>18 DE-miRNAs<br>15 DE-miRNAs	Genes involved in cellular metabolism, ligand–receptor interaction, cell adhesion, signal transduction, mediation of endocytosis and the actin cytoskeleton	(181)
		Broiler embryos	↑ miR-133<br><br>↓ miR-199a-5p, miR-1915 and miR-638, let-7, miR-93 and miR-130c	Cell maintenance and proliferation and organismal and tissue development	(182)
	Environmental pollutants: cadmium chloride	Spleen	↓ miR-33-5q	AKT/mTOR signaling and HSP70 NF-κB, p-JNK/JNK and genes involved in autophagy	(184)
		Kidney	↓miR-30a	↑ IRE-1-JNK pathway	(185)
	Environmental pollutants: lead	Neutrophils	↑ miR-16-5p	PiK3R1 and IGFR1	(186)
	Environmental pollutants: ammonia	Spleen	↓miR-6615, miR-133a	Genes involved in inflammatory response	(188)
		Heart	↓miR-202-5p	PTEN/AKT/mTOR pathway	(189)
		Splenic lymphocytes	↑ miR-15a	BCL2	(190)
	Road transportation	Serum	↑miR-22, miR-155 and miR-365	NR	(192)
